# Outer Membrane Vesicles Derived From *Fusobacterium nucleatum* Trigger Periodontitis Through Host Overimmunity

**DOI:** 10.1002/advs.202400882

**Published:** 2024-10-30

**Authors:** Li Zhang, Demao Zhang, Chengcheng Liu, Boyu Tang, Yujia Cui, Daimo Guo, Mengmeng Duan, Ying Tu, Huiling Zheng, Xinjie Ning, Yang Liu, Haoran Chen, Minglei Huang, Zhixing Niu, Yanfang Zhao, Xiaoheng Liu, Jing Xie

**Affiliations:** ^1^ State Key Laboratory of Oral Diseases & National Center for Stomatology & National Clinical Research Center for Oral Diseases, West China Hospital of Stomatology Sichuan University Chengdu Sichuan 610041 China; ^2^ Institute of Biomedical Engineering, West China School of Basic Medical Sciences and Forensic Medicine Sichuan University Chengdu 610041 China; ^3^ Department of Pediatric Dentistry, School of Dentistry University of Alabama Birmingham Birmingham 35233 USA

**Keywords:** alveolar bone, fusobacterium nucleatum, immunity, outer membrane vesicles, periodontitis

## Abstract

The virulent bacteria‐induced host immune response dominates the occurrence and progression of periodontal diseases because of the roles of individual virulence factors from these pathogens in the initiation and spread of inflammation. Outer membrane vesicles (OMVs) as a pathogenic entity have recently attracted great attention as messenger bridges between bacteria and host tissues. Herein, the novel role of OMVs derived from *Fusobacterium nucleatum* in the occurrence of periodontitis is dissected. In a rat periodontitis model, it is found that OMVs derived from *F. nucleatum* caused deterioration of periodontitis by enhancing inflammation of the periodontium and absorption of alveolar bone, which is almost equivalent to the effect of *F. nucleatum* itself. Furthermore, that OMVs can independently induce periodontitis is shown. The pathogenicity of OMVs is attributed to multiple pathogenic components identified by omics. After entering human periodontal ligament stem cells (hPDLSCs) by endocytosis, OMVs activated NLRP3 inflammasomes and impaired the mineralization of hPDLSCs through NF‐κB (p65) signaling, leading to the final injury of the periodontium and damage of alveolar bone in periodontitis. These results provide a new understanding of OMVs derived from pathogens and cues for the prevention of periodontitis.

## Graphical abstract

Xiaoheng Liu, Jing Xie and co‐workers dissect the novel role of OMVs derived from *F. nucleatum* in the occurrence of periodontitis. The severity of periodontitis caused by OMVs is close to the effect of *F. nucleatum* itself, due to the activations of NLRP3 inflammasomes, inflammatory factors, and extracellular degradation. This finding provides a new understanding of OMVs in bacteria‐host interactions.

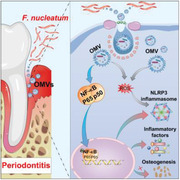

## 1 Introduction

Periodontitis is characterized as a chronic inflammatory disease that infects the surrounding tissues, including the gingiva, periodontal ligament, and alveolar bone, that support and stabilize the teeth.^[^ ^1^, ^2^ ^]^ Its global prevalence ranges from 20% to 50% and shows a continuously increasing trend.^[^ ^3^ ^]^ The initiation and progression of periodontitis are triggered by dysbiosis of oral symbiotic microbiota, leading to the formation of dental plaque and host immune defense, resulting in persistent long‐term inflammation.^[^ ^4^, ^5^ ^]^ Periodontitis at a late stage, accompanied by further loss of gingival, periodontal ligament and alveolar bone, forms the deep periodontal “pocket”, ultimately leading to intense loosening and even loss of tooth.^[^ ^1^, ^6^ ^]^ Various periodontal pathogens are involved in the dysbiosis of oral symbiotic microbiota, the most important of which are *Porphyromonas gingivalis*, *Treponema denticola*, *Tannerella forsythia*, *Fusobacterium nucleatum* and *Aggregatibacter actinomycetemcomitans*.^[^ ^7^ ^]^ These pathogens often gather and colonize the periodontal zone, secrete lipopolysaccharides (LPS), exopolysaccharides, gingipains, and leukotoxin,^[^ ^8^ ^]^ and contribute to the initiation and progression of periodontitis.^[^ ^9^ ^]^ Moreover, the products of these pathogens and even the pathogens themselves are potentially associated with systemic diseases, including neuropathy,^[^ ^10^ ^]^ cardiovascular disease,^[^ ^11^ ^]^ type 2 diabetes mellitus,^[^ ^12^ ^]^ rheumatoid arthritis (RA)^[^ ^13^ ^]^ and cancers.^[^ ^14^ ^]^

*F. nucleatum* is a gram‐negative, specialized anaerobic bacterium with a fusiform rod shape and is directly associated with the occurrence and progression of diseases such as atherosclerosis (AS),^[^ ^15^ ^]^ adverse pregnancy outcomes (APOs)^[^ ^16^ ^]^ inflammatory bowel disease (IBD),^[^ ^17^ ^]^ Alzheimer's disease (AD),^[^ ^18^ ^]^ and various types of cancers.^[^ ^7^, ^19^ ^]^ Evidence that interprets its role in these diseases comes from two aspects: colonization and invasion. *F. nucleatum* can enter circulation and lead to transient bacteremia through direct daily contact, including ulcerating gingival epithelium, accidental bleeding from brushing, and inevitable bleeding during dental treatment procedures.^[^ ^20^, ^21^ ^]^ *F. nucleatum* can also colonize the intestinal mucosa via the digestive tract.^[^ ^22^, ^23^ ^]^ Moreover, *F. nucleatum* helps promote the colonization of other pathogens, including *peptostreptococcus spp*., *leptotrichia spp*, and *campylobacter spp*. by establishing an efficient biofilm.^[^ ^24^, ^25^, ^26^ ^]^ The second is the host immune response. *F. nucleatum* triggers host immune defense by invading cells including fibroblasts, osteoblasts, cementoblasts, and immune cells via the release of virulence factors such as LPS, FadA, and Fap2, and damaging tissues through upregulation of cytokines/chemokines, including IL‐6, IL‐8, TNF‐α, CCL2, and CXCl1.^[^ ^27^, ^28^, ^29^ ^]^ In oral diseases, *F. nucleatum* is found in periodontal pockets, plaque, and infected root canals and is associated with pulpitis, periapical infections, peri‐implantitis, and various degrees of periodontal disease, such as gingivitis, chronic periodontitis, and aggressive periodontitis.^[^ ^30^, ^31^ ^]^ The detection rate and abundance of *F. nucleatum* positively correlated with the progression and deterioration of periodontal disease lesions. *F. nucleatum* is found in the deep periodontal pockets of patients with periodontitis^[^ ^32^ ^]^ and severe alveolar bone loss is associated with high colonization of *F. nucleatum*.^[^ ^33^ ^]^ Meanwhile, one report indicated that the growth inhibition of *F. nucleatum* showed a good outcome in the prevention of periodontitis deterioration.^[^ ^26^ ^]^ However, the function and underlying biomechanism of *F. nucleatum* in the initiation and progression of periodontitis require further investigation.

A completely novel field referred to bacterial extracellular vesicles (bEVs) has recently attracted our attention due to its close correlation with periodontitis. Bacterial extracellular vesicles (bEVs) are spherical double‐membrane nanoparticles harboring specific subsets of bioactive proteins, polysaccharides, lipids, and nucleic acids.^[^ ^34^, ^35^ ^]^ Outer membrane vesicles (OMVs) belong to a type of bEVs derived from gram‐negative bacterial species.^[^ ^36^ ^]^ Current reports on OMVs are very limited, but they potentially indicate the importance of OMVs in periodontal diseases because of their functions in virulence, invasion, membrane fusion, biofilm formation, phage infection, horizontal transfer of DNA, and diffusion of drug resistance.^[^ ^37^ ^]^ OMVs derived from *hypervirulent Klebsiella pneumoniae* (hvKP) generate varying degrees of toxic effects on different cell types and can cause inflammatory reactions in host cells by secreting IL‐6 and IL‐8.^[^ ^38^ ^]^ The protein hemolysin carried by OMVs from *Enterohemorrhagic Escherichia coli* can target mitochondria, induce mitochondrial dysfunction and cause apoptosis of endothelial and epithelial cells.^[^ ^39^ ^]^ Furthermore, some evidence has emphasized that bacterial OMVs might exhibit stronger toxicity than their mother bacteria due to the higher concentrations of virulence factors released during host‐bacteria interactions.^[^ ^40^ ^]^ For example, OMVs from *P. gingivalis* induce macrophages to produce large amounts of inflammatory factors (TNF‐α, IL‐12p70, IL‐6, IL‐10, and IFN‐β), which are much higher than those produced by macrophages infected with *P. gingivalis* itself.^[^ ^41^ ^]^ As to *F. nucleatum*, Hong et al. reported that *F. nucleatum* OMVs containing the virulence determinant FadA could translocate into the joints, trigger local inflammatory responses and aggravate RA.^[^ ^42^ ^]^ Engevik et al. showed that *F. nucleatum* could enhance the proinflammatory response and activate upstream signaling cascades in the context of a depleted intestinal microbiome through the delivery of OMVs, thus showing a great impact on the gut microbiome.^[^ ^43^ ^]^ Lin et al. elucidated that the OMVs from *F. nucleatum* influenced the mitochondrial fusion and promoted cell invasion of colorectal cancer cells, implying a potential complementary chemotherapy for colorectal cancer.^[^ ^44^ ^]^ However, it is unclear whether OMVs derived from *F. nucleatum* play an independent role in the occurrence and development of periodontitis.

In this study, we aimed to explore the role of OMVs derived from *F. nucleatum* in the occurrence and development of periodontitis. Herein, we isolated and characterized OMVs from *F. nucleatum*, and analyzed their proteomic characteristics of *F. nucleatum* OMVs. In the ligation‐induced rat periodontitis model, we evaluated the pathogenic independence of *F. nucleatum* OMVs by characterizing the periodontal ligament inflammation and alveolar bone loss. In vitro, we elucidated its pathogenicity pathway in human periodontal ligament stem cells (hPDLSCs) from endocytosis, transcriptome regulation, intracellular signaling, and inflammatory activation to osteogenic differentiation and mineralization.

## 2 Results

### 2.1 OMVs Derived from *F. nucleatum* are the Important Stimulator for Alveolar Bone Loss in *F. Nucleatum*‐Exacerbated Periodontitis

**Figure 1:**
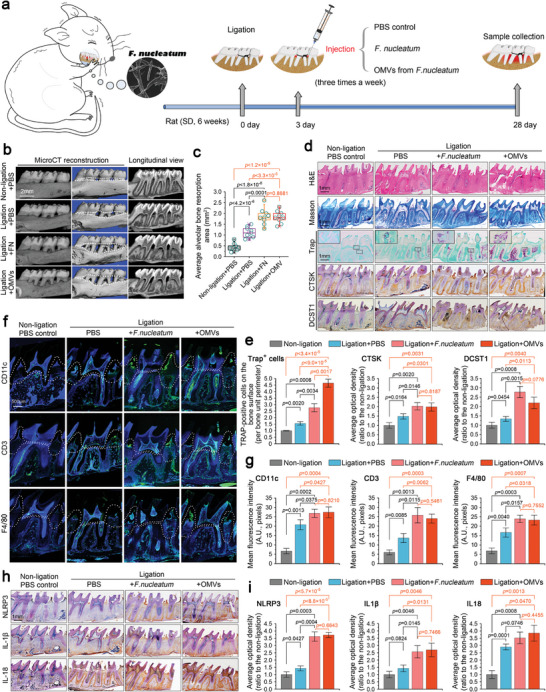
OMVs derived from *F. nucleatum* were the important stimulator for alveolar bone loss in *F.nucleatum*‐exacerbated rat periodontitis model. a) Schematic diagram showing the construction of a rat model of periodontitis through ligation, the detailed procedure of *F. nucleatum* and its OMVs administration, and alveolar bone collection. The four groups were set as follows: non‐ligation + PBS (normal control), ligation + PBS (periodontitis control), ligation + *F.nucleatum* and ligation + *F. nucleatum* OMVs. b) Micro‐CT images of the rat maxilla indicating that the effect of *F. nucleatum* OMVs in promoting alveolar bone loss was already close to that of *F. nucleatum*. Images were obtained from six independent experiments (n = 6). c) Quantitative analysis of alveolar bone resorption area in (b). Data were based from six independent experiments (n = 6). d) Histological evaluation based on H&E, Masson, and TRAP staining, and immunohistochemical evaluation of CTSK and DCST1 indicating the destruction of alveolar bone induced by F. nucleatum and F. nucleatum OMVs in a rat periodontitis model. Images were obtained from six independent experiments (n = 6). e) Quantitative analysis of Trap+ cells and the protein expression of CTSK and DCST1 in the alveolar bone of periodontitis induced by *F. nucleatum* and *F. nucleatum* OMVs in (d). Data were based from six independent experiments (n = 6). f) Immunofluorescence images showing the increase in CD11c, CD3, and F4/80 in the periodontal tissue of periodontitis induced by *F. nucleatum* and *F. nucleatum* OMVs. Images were obtained from six independent experiments (n = 6). g) Quantitative analysis based on fluorescence optical density showing an increase in CD11c, CD3 and F4/80 in (f). Data were obtained from six independent experiments (n = 6). h) Immunohistochemical images showing the increased expression of the NLRP3 inflammasome and its effectors, IL‐1β and IL‐18, in the periodontal tissue of periodontitis induced by F. nucleatum and F. nucleatum OMVs. Images were obtained from six independent experiments (n = 6). i) Quantitative analysis based on fluorescence optical density showing an increase in NLRP3, IL‐1β, and IL‐18 in (f). Data were analyzed from six independent experiments (n = 6). Data in c are presented as boxes (from 25% to 50 to 75%) and whiskers (standard deviation, SD). Data in e, g, and i are presented as mean ± SD. Data in c, e, g and i are based on Two‐tailed Student's t‐tests. Probability (p) value < 0.05 is considered statistically significant.

To determine the pathogenic role of OMVs derived from *F. nucleatum* in the deterioration of periodontitis, we first established a ligation‐induced rat periodontitis model as described previously^[^ ^45^, ^46^ ^]^ and investigated the effect of OMVs by direct injection in the periodontal zone (**Figure** **1**a). After injection for 25 days, we first evaluated the quality of the alveolar bone by using µ‐CT (Figure 1b). The results showed that *F. nucleatum* injection increased the absorption of alveolar bone in rat periodontitis, and that the injection of OMVs caused alveolar bone resorption to the extent of the injection of *F. nucleatum*. The quantification of the absorption areas confirmed this result (Figure 1c). We used histology and immunohistochemistry to detect tissue damage induced by OMVs (Figure 1d). HE and Masson staining showed that OMVs induced atrophy of the periodontal zone, which could be compared to the consequences of *F. nucleatum* induction in rat periodontitis (upper two rows). Furthermore, osteoclast activity was greatly enhanced by both *F. nucleatum* and its OMVs (middle row), and the expression of osteoclast markers, CTSK and DCST1, was increased by both *F. nucleatum* and its OMVs in rat periodontitis (lower two rows). Quantification of trap‐positive cell numbers and expression of CTSK and DCST1 confirmed the results of histology and immunohistochemistry, respectively (Figure 1e). Next, we investigated the inflammation triggered by OMVs in the periodontal zone of a rat periodontitis model (Figure 1f). The results showed that the expression of CD11c, a marker of dendritic cells;^[^ ^47^ ^]^ CD3, a marker of T cells;^[^ ^48^ ^]^ and F4/80, a marker of macrophages,^[^ ^49^ ^]^ was increased induced by *F. nucleatum* and its OMVs in rat periodontitis, indicating the initiation of a strong inflammatory reaction. Fluorescence quantification confirmed that the increase in these proteins caused by OMVs was equivalent to that caused by *F. nucleatum* (Figure 1g). Finally, we examined the inflammatory factors induced by *F. nucleatum* OMVs in the periodontal zone of rat periodontitis model (Figure 1h; Figure S1a, Supporting Information). The results revealed that the expression of NLRP3, IL‐1β, IL‐18, IL‐6, and TNF‐α was upregulated by *F. nucleatum* and its OMVs in rats with periodontitis. Quantification further confirmed that OMVs caused the expression of these inflammatory factors achieved the effects caused by *F. nucleatum* (Figure 1i; Figure S1b, Supporting Information). Meanwhile, the high expression levels of NLRP3, IL‐1β, and IL‐18 (Figure 1i) as well as those of IL‐6 and TNF‐α in Figure S1b (Supporting Information) revealed the activation of NLRP3‐mediated inflammasomes in the periodontium induced by *F. nucleatum* OMVs.^[^ ^50^ ^]^ Additionally, we used immunofluorescence to detect enhanced expression of MMP‐2 and MMP‐9, which are important indicators of tissue degradation, in the periodontal zone of a rat periodontitis model induced by OMVs (Figure S2, Supporting Information). Taken together, these results indicate that OMVs exacerbated periodontal tissue atrophy and alveolar bone resorption in the deterioration of periodontitis, almost achieving the same effect as *F. nucleatum*.

### 2.2 OMVs Derived from *F. Nucleatum* Independently Induce the Occurrence of Periodontitis

**Figure 2:**
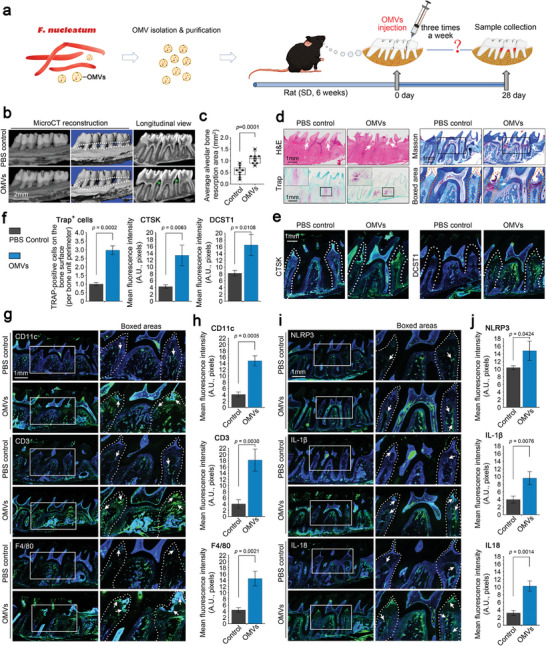
OMVs derived from *F. nucleatum* independently induced the occurrence of periodontitis and loss of alveolar bone. a) Schematic diagram showing the detailed procedure of the application of OMVs derived from *F. nucleatum* to the periodontal zone of normal rats and alveolar bone collection. b) Micro‐CT images showing the effect of *F. nucleatum* OMVs on alveolar bone loss. Images were obtained from six independent experiments (n = 6). c) Quantitative analysis of alveolar bone resorption area in (b). Data were based from six independent experiments (n = 6). d) Histological images based on H&E, Masson, and TRAP staining showing the effect of *F. nucleatum* OMVs on alveolar bone loss. Images were obtained from six independent experiments (n = 6). e) Immunofluorescence images showing increased expression of CTSK and DCST1 in periodontal tissue induced by *F. nucleatum* OMVs. Images were obtained from six independent experiments (n = 6). f) Quantitative analysis of Trap+ cells in (d) and the protein expression of CTSK and DCST1 in (e) in periodontal tissue induced by *F. nucleatum* OMVs. Data were based on six independent experiments (n = 6). g) Immunofluorescence images showing the increase in CD11c, CD3 and F4/80 in periodontal tissue induced by *F. nucleatum* OMVs. Images were obtained from six independent experiments (n = 6). h) Quantitative analysis of CD11c, CD3, and F4/80 in periodontal tissue induced by *F. nucleatum* OMVs in (g). Data were obtained from six independent experiments (n = 6). i) Immunofluorescence images showing increased expression of NLRP3 inflammasome and its effectors, IL‐1β and IL‐18, in the periodontal tissue induced by *F. nucleatum* OMVs. Images were obtained from six independent experiments (n = 6). j) Quantitative analysis based on fluorescence optical density showing an increase in NLRP3, IL‐1β and IL‐18 levels in (i). Data were obtained from six independent experiments (n = 6). Data in c are presented as boxes (from 25% to 50 to 75%) and whiskers (standard deviation, SD). Data in f, h, and j are presented as mean ± SD. Data in c, f, h, and j are based on Two‐tailed Student's t‐tests. Probability (p) value < 0.05 is considered statistically significant.

To further investigate the role of OMVs derived from *F. nucleatum* in the occurrence of periodontitis, we directly injected purified OMVs into the normal periodontal tissue of rats (**Figure** **2**a). After injection for 25 days, we first evaluated the change in the alveolar bone by using µ‐CT (Figure 2b). These results indicated that OMVs induced alveolar bone resorption. Quantification of the average bone loss areas confirmed these results (Figure 2c). We used histology and immunofluorescence to detect damage to the periodontal zone induced by OMVs (Figure 2d,e). HE staining showed that OMVs caused tissue fragmentation in the entire periodontal zone, and Masson staining further indicated that many fibrous tissues were generated in these tissue fragments. TRAP staining revealed higher activity of osteoclasts in the periodontal zone induced by OMVs (Figure 2d). Immunofluorescence of CTSK and DCST1 (Figure 2e) showed their higher expression in the periodontal zone induced by OMVs. Quantification of Trap‐positive cells and the expression of CTSK and DCST1 confirmed these results (Figure 2f). Next, we detected the recruitment of inflammatory cells by characterizing the expression of CD11c, CD3, and F4/80 induced by OMVs (Figure 2g). Immunofluorescence results showed that the expression of these protein markers was all enhanced in both the periodontal zone and alveolar bone area induced by OMVs. Quantification of immunofluorescence intensity confirmed the fold‐changes in these proteins (Figure 2h). Finally, we investigated the changes in the inflammatory factors induced by OMVs (Figure 2i; Figure S3, Supporting Information). Using immunofluorescence, we found that OMVs activated NLRP3 inflammasomes by inducing higher expressions of NLRP3, IL‐1β, and IL‐18 (Figure 2i and j). Additionally, we found that the inflammatory factors IL‐6 and TNF‐α were significantly enhanced in the periodontal zone induced by OMVs (Figure S3, Supporting Information). Collectively, these results indicated that OMVs derived from *F. nucleatum* could independently trigger the occurrence of periodontitis.

### 2.3 Characterization of OMVs Derived from *F. Nucleatum* Decodes the Landscape of Its Pathogenicity

**Figure 3:**
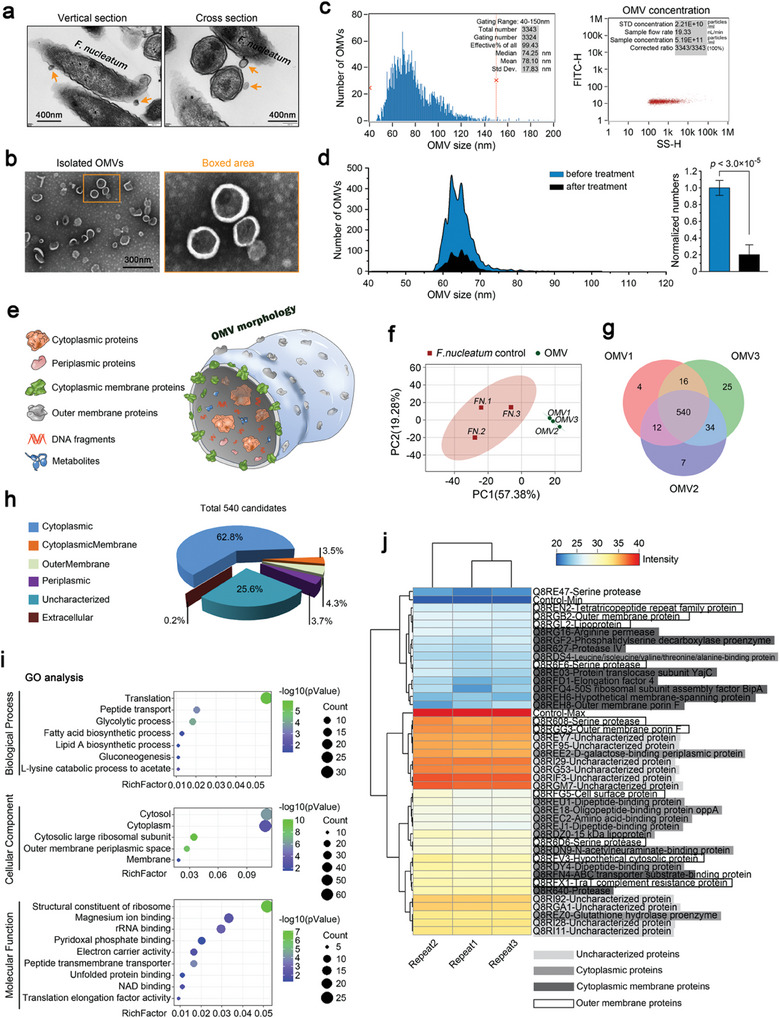
The characterization of OMVs derived from *F. nucleatum*. a) TEM images showing the morphologies of OMVs secreted by *F. nucleatum* through the vertical section (left) and cross‐sections (right) in its natural state. Images were selected based on eight independent experiments (n = 8). b) TEM images showing the morphology of OMVs isolated from *F. nucleatum*. Images were obtained from four independent experiments (n = 4). c) Nanoflow cytometry indicating the particle size (left) and number (right) of OMVs collected from *F.nucleatum*. Images were based from three independent experiments (n = 3). d) Treatment with Triton X‐100 indicating the purity of OMVs collected from *F. nucleatum*. The application of Triton X‐100 destroyed the membrane structure of the OMVs. The differences in the detection of particle number before (blue) and after (black) Triton X‐100 treatment reflected the OMV purity. Histogram (right) is presented as mean ± SD. Probability (p) value < 0.05 is considered statistically significant. e) Schematic diagram indicating the structure of OMVs isolated from *F. nucleatum*. f) Principal component analysis (PCA) of proteomics indicating the similarity between three independent samples of *F.nucleatum* and *F. nucleatum* OMVs. Samples of *F. nucleatum* were used as the controls. g) Venn diagram indicating the number of shared proteins obtained from three independent *F. nucleatum* OMVs. h) Pie chart showing the composition and proportion of the shared 540 proteins in OMVs derived from *F.nucleatum*. i) GO analysis showing the enrichment of these shared 540 proteins of OMVs derived from *F. nucleatum* in biological process (BP), cellular component (CC), and molecular function (MF). j) Pheatmap showing the top 10 proteins in the outer membrane, cytoplasmic membrane, cytoplasm, and uncharacterized candidates. The range of expression intensity was 20–40, and the pheatmap was presented directly as the intensity value. The minimum expression intensity “20” was set as “control min” and the maximum expression intensity “40” was set as “control max”. The serine protease (Q8RE47) with the expression intensity “1” was set as a background control group.

Before we decoded the protein components of OMVs, we first characterized OMVs derived from *F. nucleatum*. We isolated and purified OMVs from the BHI media of *F. nucleatum* through ultrafiltration and ultracentrifugation, as previously described^[^ ^42^, ^43^ ^]^ (Figure S4a, Supporting Information). The isolated OMVs showed typical vesicular features in SEM (Figure S4b, Supporting Information). SDS‐PAGE was used to show the total protein profile of OMVs relative to that of *F. nucleatum* (Figure S4c,d, Supporting Information). By density gradient ultracentrifugation, we finally chose to collect OMVs at 15%–25% layers for all experiments in the study (Figure S4e, Supporting Information). We first observed the morphology and size of OMVs secreted by *F. nucleatum* in their natural state using TEM (**Figure** **3**a). Following density gradient ultracentrifugation, we obtained a large number of OMVs. These OMVs were membrane‐encapsulated spherical vesicles of varying sizes (Figure 3b). Using nanoflow cytometry, we showed the particle size map of the isolated OMVs (Figure 3c). The data indicated that the average diameter of OMVs purified from *F. nucleatum* was 74.25 nm ranged from 40 to 150 nm. The data also indicated that OMVs could be collected at high concentrations (up to 5.19E+11 particles per 1 ml). To further demonstrate that the OMVs obtained were nanoparticles with intact vesicular membranes, we treated OMVs with Triton X‐100 as described previously.^[^ ^51^ ^]^ The results of nanoflow cytometry showed that Triton X‐100 treatment dissolved up to 80% of OMV nanoparticles (Figure 3d), indicating that the OMVs we isolated had a highly intact membrane structure.

As the diverse protein components of OMVs play a core role in their pathogenicity^[^ ^52^ ^]^ (Figure 3e), we used protein mass spectrometry (MS) to decode the total protein profile of OMVs isolated from *F. nucleatum*. Using *F. nucleatum* as a control, the results based on principal component analysis (PCA) showed that the similarity among the independent OMV samples was very high (Figure 3f). From these three independent OMVs, 540 co‐expressed protein candidates were identified using a Venn diagram (Figure 3g). Proteomic analysis did not identify cow or calf proteins, indicating that the purified *F. nucleatum* OMVs used in this study were free of potential contaminants from the culture media. We divided these co‐expressed proteins into six subtypes, that is, cytoplasmic, cytoplasmicmembrane, outermembrane, periplasmic, extracellular and uncharacterized proteins, based on their distribution positions, and found that the subtype cytoplasmic proteins, ranked first (62.8%) and the subtype uncharacterized proteins ranked second (25.6%). The proportion of other subtypes was relatively small (cytoplasmic membrane proteins, 3.5%; outermembrane proteins, 4.3%; periplasmic proteins, 3.7%) (Figure 3h). Through GO analysis, we identified the roles of these proteins in biological processes (BP), cell component (CC) and molecular function (MF) (Figure 3i). Importantly, we clustered the top 10 proteins in the four major subtypes including cytoplasmic, uncharacterized, cytoplasmicmembrane and outermembrane by pheatmap (Figure 3j). Among them, proteins, such as serine protease,^[^ ^53^, ^54^ ^]^ tetratricopeptide repeat family protein,^[^ ^55^ ^]^ outer membrane protein‐Q8RGB2,^[^ ^56^ ^]^ lipoprotein,^[^ ^57^ ^]^ outer membrane porin F,^[^ ^58^ ^]^ and ABC transporter substrate‐binding protein,^[^ ^59^ ^]^ have been reported to be correlated with the pathogenicity of periodontitis.

### 2.4 OMVs Derived from *F. Nucleatum* Triggers NLRP3 Inflammasomes and Impair the Mineralization Through Activation of NF‐κB Signaling

**Figure 4:**
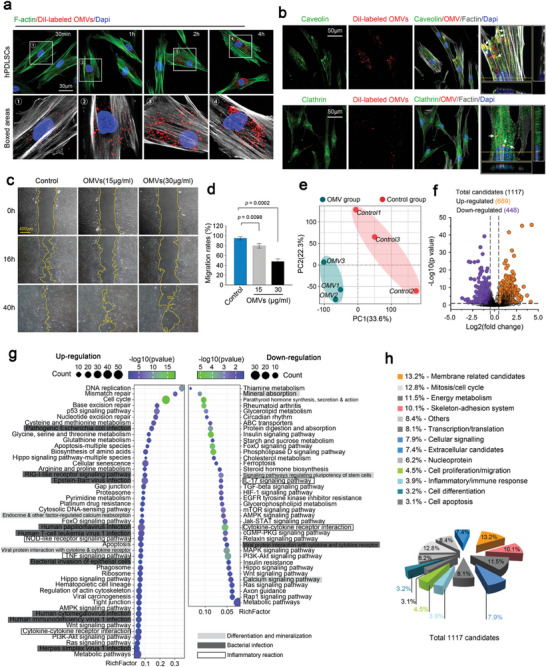
OMVs derived from *F. nucleatum* triggered the gene profile perturbation of hPDLSCs on transcriptome level. a) Fluorescent staining showing endocytosis of DiI‐labeled OMVs by PDLSCs. DiI‐labeled OMVs, red; F‐actin, green; nuclei, blue. Images were obtained from three independent experiments (n = 3). b) Immunofluorescence staining showing the colocalization of DiI‐labeled OMVs with the endocytosis proteins, caveolin and clathrin. DiI‐labeled OMVs, red; Caveolin and Clathrin, green; F‐actin, gray; nuclei, blue. Projection images and vertical optical sections (x‐z and y‐z planes) demonstrate the colocalization of OMVs with endocytic proteins. Images were obtained from three independent experiments (n = 3). c) Scratch wound‐healing assay showing impairment in migration of hPDLSCs induced by *F. nucleatum* OMVs. Images were obtained from three independent experiments (n = 3). d) Quantification of average migration rate in (c) at 40 h. Data were obtained from three independent experiments (n = 3) and presented as mean ± SD. Probability (p) value < 0.05 is considered statistically significant. e) Principal component analysis (PCA) of RNA sequencing indicating the similarity of three independent cell samples from normal and OMV‐treated groups. f) Volcano plot showing the altered gene candidates in PDLSCs induced by OMVs. The fold change was set to be 1.5 (OMV group versus normal group) and the p value was set to 0.05. Purple indicates downregulated gene candidates and orange indicates upregulated gene candidates. g) KEGG analysis indicating the pathways involving bacterial infection, inflammatory reactions and differentiation/mineralization of PDLSCs induced by *F. nucleatum* OMVs. (f) Pie chart showing the proportion of all changed gene candidates in the cellular biological processes of PDLSCs induced by *F. nucleatum* OMVs. The current study involved the processes of endocytosis (membrane‐related candidates‐13.2%, skeleton‐adhesion system‐10.1%), signaling (cellular signaling‐7.9%), inflammation (inflammatory/immune response‐3.9%), and cell responses (cell proliferation/migration‐4.5%, cell differentiation‐3.2%), which are highlighted by colored pieces of pie.

To further reveal the underlying pathogenicity of periodontitis induced by OMVs, we explored the interaction between *F. nucleatum* OMVs and host cells, hPDLSCs, which play a crucial role in the repair and regeneration of periodontal tissue.^[^ ^60^, ^61^, ^62^, ^63^ ^]^ The stemness of harvested hPDLSCs was verified (Figure S15, Supporting Information). We labeled OMVs with Dil (red) and found that OMVs could enter the cytoplasm of hPDLSCs (**Figure** **4**a; Figure S5, Supporting Information). As OMVs from gram‐negative bacteria enter host cells in several different ways, that is, caveolin‐ and clathrin‐dependent and lipid raft‐endocytosis,^[^ ^63^ ^]^ we traced Dil‐labeled OMVs and found that they co‐localized with caveolin and clathrin when they entered hPDLSCs (Figure 4b). Importantly, their colocalization intensity with caveolin was much higher than that of clathrin. We used methyl‐β‐cyclodextrin, an inhibitor that interferes with cholesterol and damages lipid rafts and inhibits lipid raft‐dependent and caveolin‐dependent endocytosis;^[^ ^64^ ^]^ dynasore, an inhibitor of dynamin that could inhibit dynamin‐related clathrin‐dependent and caveolin‐dependent endocytosis;^[^ ^65^ ^]^ and chlorpromazine, an inhibitor of clathrin‐mediated endocytosis,^[^ ^66^ ^]^ to compare the main pathways that OMVs enter into hPDLSCs (Figure S6, Supporting Information). CCK‐8 assay showed that 80 µM dynasore, 5 mM MβCD, and 15 µg ml^−1^ chlorpromazine had no obvious cytotoxicity in hPDLSCs (Figure S16, Supporting Information). The results showed that OMVs entered into the hPDLSCs through endocytosis, among which the caveolin‐dependent endocytosis was dominant (In the following data of RNA sequencing, we also confirmed the gene changes related to the caveolin‐dependent endocytosis by pheatmap (Figure S7, Supporting Information)). We then detected the cell migration and proliferation of hPDLSCs after endocytozing OMVs and found that the migration of hPDLSCs was largely reduced by the wounding healing assay (Figure 4c,d), while cell proliferation (or cell viability) was unchanged by the CCK8 assay (Figure S8, Supporting Information). To further reveal transcriptome perturbation of hPDLSCs induced by OMVs derived from *F. nucleatum*, we performed bulk RNA sequencing. After determining that the similarity of the samples analyzed by PCA made the grade (Figure 4e), we screened 29829 genes and found 1117 differentially expressed genes (669 upregulated and 448 downregulated) using a volcano plot (Figure 4f, fold change > 1.5, p vaule < 0.05). From KEEG enrichment analysis, we clustered all pathways and found that there were three parts, that is, bacterial infection, inflammatory reaction, and differentiation and mineralization, with an absolutely high proportion among these pathways (Figure 4g). We classified and listed all proportions of these genes according to their functions and cellular locations (Figure 4h) and found that these three parts in Figure 4g participated in the regulation of membrane‐related candidates, skeleton‐adhesion system, cellular signaling, inflammatory/immune response, cell proliferation/migration, extracellular secretion to cell differentiation (colored subclasses), indicating their importance in mediating the cell behaviors of hPDLSCs.

**Figure 5:**
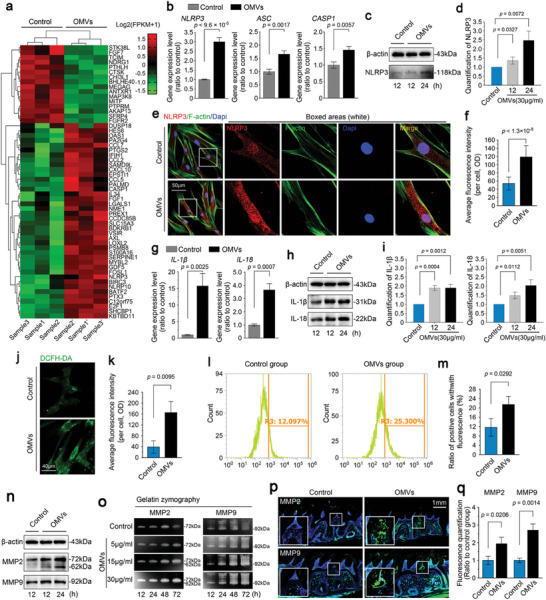
OMVs derived from *F. nucleatum* activated NLRP3 inflammasome from the upstream initiator to downstream inflammatory factors and proteolytic enzymes. a) Pheatmap based on RNA sequencing indicating the gene candidates involved in inflammation and differentiation of hPDLSCs induced by *F. nucleatum* OMVs. All gene data are presented as log2 (FPKM +1). Cell samples 1 & 1′, 2 & 2′, and 3 & 3′ were isolated from the same donators. b) qPCR confirmed the expression of NLRP3, ASC, and CASP1 in hPDLSCs induced by *F. nucleatum* OMVs. Data were presented as three independent experiments (n = 3). c) Western blotting showing the protein expression of the NLRP3 inflammasome in hPDLSCs induced by *F. nucleatum* OMVs. Images were obtained from three independent experiments (n = 3). d) Quantitative analysis further confirmed the protein changes in (c). Data were obtained from three independent experiments (n = 3). e) Immunofluorescence staining showing increased expression of the NLRP3 inflammasome in hPDLSCs induced by *F. nucleatum* OMVs. Images were obtained from three independent experiments (n = 3). f) Quantitative analysis of the fluorescence optical density further confirmed the protein changes in (e). Data were obtained from three independent experiments (n = 3). g) qPCR showing the expression of IL‐1β and IL‐18 in hPDLSCs induced by *F. nucleatum* OMVs. Data were presented as three independent experiments (n = 3). h) Western blotting showing the protein expression of IL‐1β and IL‐18 in hPDLSCs induced by *F. nucleatum* OMVs. Images were presented as three independent experiments (n = 3). i) Quantitative analysis of IL‐1β and IL‐18 protein levels in (h). Data were obtained from three independent experiments (n = 3). j) Fluorescent staining showing changes in ROS levels in hPDLSCs induced by *F. nucleatum* OMVs. Images were presented as three independent experiments (n = 3). k) Quantitative analysis of the fluorescence optical density in (j). Data were obtained from three independent experiments (n = 3). l) Flow cytometry showing changes in ROS levels in hPDLSCs induced by *F. nucleatum* OMVs. Data were presented as three independent experiments (n = 3). m) Quantitative analysis of fluorescence‐positive cells in (l). Data were obtained from three independent experiments (n = 3). n) Western blotting showing changes in the expression of MMP2 and 9 in hPDLSCs induced by *F. nucleatum* OMVs. Images were presented as three independent experiments (n = 3). o) Gelatin zymography showing the activities of MMP2 and 9 in hPDLSCs induced by *F. nucleatum* OMVs. The gels were subjected to three independent experiments (n = 3). p) Immunofluorescence staining indicating changes in the expression of MMP2 and 9 in periodontal tissue induced by *F. nucleatum* OMVs. q) Quantitative analysis of the fluorescence optical density further confirmed the expression changes in (p). The data were obtained from three independent experiments (n = 3). Data in b, d, f, g, i, k, m, and q are presented as mean ± SD, and all significant differences in b, d, f, g, i, k, m, and q are based on based on Two‐tailed Student's t‐tests. Probability (p) value < 0.05 is considered statistically significant.

We further analyzed the candidate genes involved in the inflammatory reaction and cell differentiation using KEGG enrichment (Figure 4g) and clustered them in a pheatmap (**Figure** **5**a). We noticed that genes related to NLRP3 inflammasomes, including NLRP3 itself and caspase1 (CASP1), were upregulated. To confirm these changes, we used qPCR to detect the expression of NLRP3, CASP1, and other inflammation‐related genes, including apoptosis‐associated speck‐like protein containing a CARD (ASC), IL‐6, IL‐8, TNF‐α, and COX2. The results indicated that the expression of NLRP3, ASC, and CASP1, the major components of NLRP3 inflammasomes,^[^ ^50^, ^67^ ^]^ was greatly increased (Figure 5b), and the other related gene expressions of IL‐6, IL‐8, TNF‐α, and COX2 were also significantly enhanced (Figure S9, Supporting Information). At the protein level, we detected the expression of NLRP3 by western blotting, and the results showed that OMVs could enhance its expression in hPDLSCs (Figure 5c,d). To further investigate the intracellular distribution of NLRP3, we used immunofluorescence and observed that its protein expression level was upregulated throughout the cell (Figure 5e,f). We then detected the downstream inflammatory molecules of the NLRP3 inflammasome. We showed the expression of IL‐1β and IL‐18, which are the direct downstream effectors of NLRP3 inflammasomes,^[^ ^67^ ^]^ at the gene and protein levels (Figure 5g–i) and found that the gene and protein expression of IL‐1β and IL‐18 were greatly enhanced by qPCR (Figure 5g) and western blotting (Figure 5h and i), respectively. Moreover, the inflammatory factors, IL‐6 and TNF‐α, were all up‐regulated at the protein level (Figure S10, Supporting Information). We next detected the reactive oxygen species (ROS), as a crucial upstream initiator of NLRP3 inflammasomes.^[^ ^68^ ^]^ We detected the activation of ROS in hPDLSCs induced by OMVs through DCFH‐DA staining (Figure 5j,k) and confirmed its higher expression levels in hPDLSCs after treatment with OMVs using flow cytometry (Figure 5i and m; Figure S11, Supporting Information). Finally, we examined the extracellular matrix degrading enzymes, MMP2 and 9, triggered by NLRP3 inflammasomes (Figure 5n–q; Figures S12 and S13, Supporting Information). By western blotting, we found that the protein expressions of MMP2 and 9 were increased in hPDLSCs after treatment with OMVs (Figure 5n; Figure S12, Supporting Information). Using gelatin zymography, we further confirmed that the protein activities of MMP2 and 9 were significantly enhanced in OMV‐induced hPDLSCs (Figure 5o; Figure S13, Supporting Information). Moreover, in rat periodontitis, we used immunofluorescence and found that the expression of MMP2 and 9 was higher in both the periodontal zone and alveolar bone area after OMVs injection (Figure 5p,q). Collectively, these results indicate that OMVs induce the activation of NLRP3 inflammasomes from the upstream initiator to downstream inflammatory factors and proteolytic enzymes in hPDLSCs.

**Figure 6:**
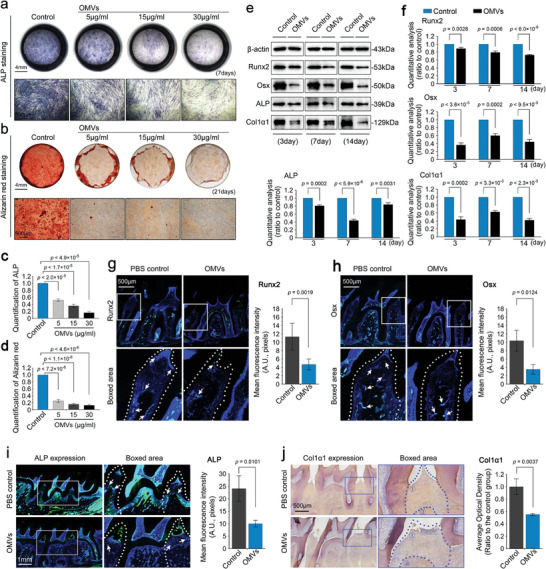
OMVs derived from *F. nucleatum* impaired mineralization capacity of hPDLSCs. a) ALP staining showing changes in osteogenic differentiation of hPDLSCs induced by *F. nucleatum* OMVs. hPDLSCs were induced by *F. nucleatum* OMVs in osteogenic induction medium for 7 days. Images were presented as three independent experiments (n = 3). b) Alizarin red staining showing changes in mineralization of hPDLSCs induced by *F. nucleatum* OMVs. hPDLSCs were induced with *F. nucleatum* OMVs in osteogenic induction medium for 21 days. Images were presented as three independent experiments (n = 3). c) Quantitative analysis of ALP staining in (a) confirming the differences in osteogenic differentiation. Data were presented as three independent experiments (n = 3). d) Quantitative analysis of alizarin red staining in (b) confirming the differences in cell mineralization. Data were presented as three independent experiments (n = 3). e) Western blotting showing the protein changes in Runx2, Osx, ALP, and Colα1 in hPDLSCs induced by *F.nucleatum* OMVs in osteogenic induction media for 3, 7, and 14 days. Images were presented as three independent experiments (n = 3). f) Quantitative analysis of Runx2, Osx, ALP, and Colα1 in (e). Data were presented as three independent experiments (n = 3). g) Immunofluorescence staining showing changes in the expression of Runx2 in periodontal tissue induced by *F.nucleatum* OMVs. Immunofluorescence images (left) and quantification (right) were presented based on three independent experiments (n = 3). h) Immunofluorescence staining showing changes in the expression of Osx in periodontal tissue induced by *F.nucleatum* OMVs. Immunofluorescence images (left) and quantification (right) were presented based on three independent experiments (n = 3). i) Immunofluorescence staining showing changes in the expression of ALP in periodontal tissue induced by *F.nucleatum* OMVs. Immunofluorescence images (left) and quantification (right) were presented based on three independent experiments (n = 3). j) Immunofluorescence staining showing changes in the expression of Col1α1 in periodontal tissue induced by *F.nucleatum* OMVs. Immunofluorescence images (left) and quantification (right) were presented based on three independent experiments (n = 3). Data in c, d, f, g, h, i, and j are presented as mean ± SD, and all significant differences in c, d, f, g, h, i, and j are based on based on Two‐tailed Student's t‐tests. Probability (p) value < 0.05 is considered statistically significant.

Activation of the NLRP3 inflammasome disrupts tissue homeostasis, making the tissue prone to degradation and destruction. Herein, we wonder if OMV injection would have an impact on the mineralization capacity of hPDLSCs, which is regarded as a vital step in periodontal tissue repair and regeneration.^[^ ^5^, ^61^ ^]^ In vitro, we examined the osteogenic differentiation and mineralization of hPDLSCs induced by OMVs in osteogenic differentiation induction media. The results showed that OMVs decreased the osteogenic differentiation capacity of hPDLSCs after induction for 7 days in a dose‐dependent manner (**Figure** **6a**,c). Moreover, after 21 days of induction, the mineralization capacity of hPDLSCs was significantly reduced by OMVs owing to the formation of extremely small amounts of calcium nodules compared to the normal group (Figure 6b,d). Using western blotting, we detected the expression of Runx2 and Osx, the transcription factors involved in osteogenic differentiation,^[^ ^69^ ^]^ and ALP and Col1α1, the regulator and substrate element in mineralization,^[^ ^70^ ^]^ respectively, and found that the expression of these proteins was all decreased in hPDLSCs induced by OMVs in osteogenic differentiation induction media within 14 days (Figure 6e,f). In OMV‐induced rat periodontitis, immunofluorescence was used to detect the expression of Runx2 (Figure 6g) and Osx (Figure 6h). The results showed that these two transcriptional factors were decreased in the alveolar bone area after OMVs injection (white arrows indicate). The expression of ALP protein in the periodontal zone and alveolar bone area after OMVs injection was greatly decreased relative to the PBS control group (Figure 6i). Additionally, using immunohistochemistry, we found that the expression of Col1α1 was significantly impaired in both the periodontal zone and alveolar bone area after OMVs injection (Figure 6j). Taken together, these results indicate that the OMV injection impairs the mineralization capacity of hPDLSCs.

**Figure 7:**
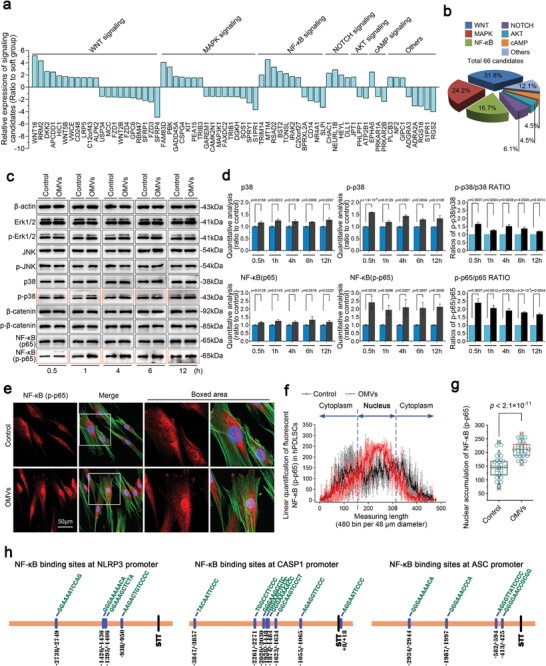
OMVs from *F. nucleatum* activated NF‐κB signaling in hPDLSCs. a) Histogram indicating the related gene changes involved in the main signaling pathways in hPDLSCs induced by *F. nucleatum* OMVs based on RNA sequencing. Data were presented as direct fold‐change (> 1.5 folds). b) Pie chart further indicating the proportion of genes in each signaling pathway in the entire pathway network. c) Western blotting showing changes in the expression of Erk1/2, JNK, p38, β‐catenin and NF‐κB (p65). Images were presented as three independent experiments (n = 3). d) Quantitative analysis of p38/p‐p38 and NF‐κB (p65/p‐p65) in (e). Data were presented as three independent experiments (n = 3) and presented as mean ± SD. Probability (p) value < 0.05 is considered statistically significant. e) Immunofluorescence staining showing the increased expression and nuclear accumulation of NF‐κB (p‐p65) in hPDLSCs induced by *F. nucleatum* OMVs. NF‐κB p‐p65, red; F‐actin, green; nuclei, blue. f) Linear quantification showing the change in NF‐κB p‐p65 level in the cytoplasmic and nuclear regions of hPDLSCs induced by *F. nucleatum* OMVs. Images represented four independent experiments (n = 4). g) Quantification of total fluorescence intensity showing the change in NF‐κB (p‐p65) in nuclear accumulation in hPDLSCs induced by *F. nucleatum* OMVs. Data were based on 35 cells from three independent experiments (n = 4) and presented as mean ± SD. Probability (p) value < 0.05 is considered statistically significant. h) Bioinformatics indicating the potential binding sites of NF‐κB at the promoters of the NLRP3, CASP1, and ASC genes.

As OMVs derived from *F. nucleatum* could both trigger inflammatory reactions and impair the mineralization capacity of hPDLSCs, we aimed to explore the signaling pathways that potentially drive these behaviors. Based on the KEGG enrichment of RNA sequencing, we screened and clustered all genes related to these signaling pathways (**Figure** **7**a). The results indicated that six main pathways were involved. The top three genes involved in the Wnt, MAPK and NF‐κB pathways collectively accounted for 72.70% of all candidate genes (Figure 7b). Thus, we focused on these three pathways and investigated their changes in hPDLSCs after treatment with OMVs within 12 h. By western blotting, we found that phosphorylated p65 (p‐p65) of NF‐κB and phosphorylated p38 (p‐p38) in MAPK signaling were activated (Figure 7c). From quantitative analysis of these signaling proteins, we confirmed that NF‐κB signaling was the pathway with the most significant changes due to the larger net increase in the expression of p‐p65 relative to other signaling proteins (Figure 7d; Figure S14, Supporting Information). Furthermore, using immunofluorescence, we observed upregulation of p‐p65 in hPDLSCs induced by OMVs for 6 h (Figure 7e). Importantly, we analyzed the increase in p‐p65 by linear immunofluorescenc quantification and found that the nuclear accumulation of p‐p65 was obvious (Figure 7f). We then quantified the total amount of p‐p65 in the nuclear regions of hPDLSCs induced by OMVs and confirmed this increase (Figure 7g). Nuclear accumulation of p‐p65 indicates that the NF‐κB signaling directly enters the nucleus and potentially regulates the gene transcriptome.^[^ ^71^, ^72^ ^]^ Thus, we used bioinformatics to establish a potential correlation between p‐p65 and the NLRP3 inflammasome (Figure 7h). From the bioinformatics, we could observe the potential binding sites of NF‐κB at the promoters of NLRP3, CASP1, and ASC referring the interaction between NF‐κB and NLRP3 inflammasomes, although NLRP3 inflammasomes also can be regarded as being upstream of NF‐κB signaling.^[^ ^73^ ^]^

To prove the important role of NF‐κB signaling in OMV‐induced NLRP3 inflammasomes and mineralization of hPDLSCs, we used SC75741, a specific NF‐κB inhibitor, to block NF‐κB signaling (Figure S17a,b, Supporting Information). Using western blotting, we found that blocking NF‐κB signaling can impair OMV‐upregulated the expression of NLRP3, IL‐18 and IL‐1β (Figure S17c,d, Supporting Information). ALP staining and Alizarin Red staining showed that blocking NF‐κB signaling partly restored OMV‐impaired ALP expression (Figure S17e, Supporting Information) and the formation of calcified nodules (Figure S17f, Supporting Information).

## 3 Discussion

**Figure 8:**
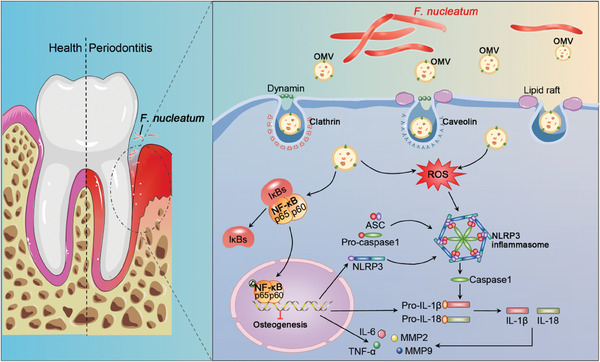
Schematic diagram showing the mechanism of OMVs from *F. nucleatum* induce periodontitis through NLRP3 inflammasomes and impair the mineralization through activation of NF‐κB signaling. PDLSCs internalize *F. nucleatum* OMVs via the endocytosis, then activate NLRP3 inflammasomes by up‐regulating the inflammatory factors and proteolytic enzymes and suppress osteogenic capacity, which involve in the participation of NF‐kB signaling.

*F. nucleatum* is considered a key pathogenic bacteria closely related to the occurrence and development of periodontitis.^[^ ^32^, ^33^ ^]^ Moreover, *F. nucleatum* contributes to systemic diseases, such as adverse pregnancy outcomes, gastrointestinal disorders, respiratory tract infections, Alzheimer's disease, cardiovascular disease, neuropathy, rheumatoid arthritis, and cancers.^[^ ^10^, ^11^, ^12^, ^13^, ^14^, ^15^, ^16^, ^17^, ^18^, ^19^ ^]^ Local injection with *F. nucleatum* in mouse with chronic periodontitis aggravates alveolar bone loss and enhances inflammatory factor secretions.^[^ ^33^, ^74^ ^]^ In this study, we focused on OMVs derived from *F. nucleatum* and systematically investigated their importance in the initiation and deterioration of periodontitis. The results indicated that the pathogenicity of *F. nucleatum* in periodontitis disease was mainly dependent on its OMVs. These results help understand the bacteria‐host cell interaction and reveal the pathogenic function of OMVs from bacteria (**Figure** **8**).

Recent reports on OMVs released by pathogenic bacteria could partially help interpret their importance in host tissue inflammation and diseases. OMVs can bypass cell‐to‐cell contact, directly allowing the release of virulence factors, such as LPS and cytokines, including interleukins, and thus participate in the crosstalk between bacteria and host cells.^[^ ^37^, ^38^, ^39^, ^40^, ^41^, ^42^, ^43^ ^]^ OMVs secreted by periodontal pathogens, including *P. gingivalis*, *T. forsythia*, have been proved to be involved in periodontal inflammatory reaction and tissue damage.^[^ ^75^ ^]^ Some reports have shown that OMVs derived from *P. gingivalis* have the capacity to invade oral epithelial cells and affect cell behavior, including cell migration and apoptosis.^[^ ^76^, ^77^ ^]^ These OMVs also induce human gingival epithelial cells and macrophages to secrete inflammatory factors, including IL‐6, IL‐8, and COX‐2.^[^ ^41^, ^78^ ^]^ Moreover, a recent study indicated that these *P. gingivalis* OMVs promoted the release of cytokines and induced the final apoptosis of hPDLSCs, which was dependent on the activation of p53 methylation.^[^ ^79^ ^]^ The OMVs derived from *T. forsythia* could also have the capacity to induce periodontal ligament fibroblasts, dendritic cells and monocytes to produce large amounts of inflammatory cytokines.^[^ ^80^, ^81^ ^]^ In gut diseases, *F. nucleatum* OMVs trigger innate immunity of human intestinal epithelial cells (hIECs) and this host immunoreaction is dependent on the participation of the protein FomA, the outer membrane protein porin in OMVs.^[^ ^82^ ^]^ *F. nucleatum* OMVs could also act as a bridge between *F. nucleatum* and human colonoid monolayers and interfere with bacterial community homeostasis in the gastrointestinal tract.^[^ ^43^ ^]^ Furthermore, in ulcerative colitis (UC), *F. nucleatum* OMVs promote epithelial barrier loss, cause oxidative stress damage, and consequently induce epithelial necroptosis via the upregulation of receptor‐interacting protein kinase 3 (RIPK3) and receptor‐interacting protein kinase 1 (RIPK1).^[^ ^83^ ^]^ A recent report indicated that *F. nucleatum* is enriched in patients with RA and is further correlated with RA severity. *F. nucleatum* OMVs carrying the virulence factor FadA can translocate into joints and trigger synovial inflammation through the activation of macrophage residents.^[^ ^42^ ^]^ In periodontitis, there was only one report of *F. nucleatum* OMVs in periodontal diseases by characterizing the role of macrophages, which indicates the polarization of macrophages toward the proinflammatory M1 phenotype and enhanced the OMV toxicity on the cell viability of gingival fibroblasts (GFs).^[^ ^74^ ^]^ In the current study, we found that *F. nucleatum* OMVs exacerbated alveolar bone resorption in rats with periodontitis, which was equivalent to the direct effect of *F. nucleatum*. The loss of alveolar bone is accompanied by the intervention of inflammatory factors and the activation of osteoclasts. Moreover, OMV injection could independently initiate the occurrence of periodontitis, which was the core discovery of this study.

The first barrier that protects against the invasion of deep periodontal tissues by periodontal pathogens is the gingival epithelial cells layers.^[^ ^62^ ^]^ Tight junctions between gingival epithelial cells ensure the integrity of the gingival epithelial barrier. The subgingival biofilm affects the desmosome junction of gingival epithelial cells, which may directly damage the structural integrity of gingival tissue, facilitating bacterial invasion and chronic infection.^[^ ^84^ ^]^ Some studies have reported that periodontal pathogens, such as *F. nucleatum* and *P. gingivalis*, showed strong invasive capacity toward gingival epithelial cells.^[^ ^85^, ^86^ ^]^ *F. nucleatum* was observed in the cytoplasm of gingival epithelial cells after co‐incubation with labeled *F. nucleatum* for 4–24 h.^[^ ^85^ ^]^ *T. denticola* invades epithelial cells via lipid raft‐mediated endocytosis, and dentilisin activity of *T. denticola* plays an important role in this process.^[^ ^87^ ^]^ Moreover, OMVs derived from these pathogens significantly contribute to the invasion and destruction of the epithelial barrier. OMVs from *P. gingivalis* are internalized by gingival epithelial cells and then degrade cellular functional molecules such as TfR and paxillin/FAK, leading to cellular dysfunction.^[^ ^77^, ^88^, ^89^ ^]^ Dentilisin‐containing OMVs from *T. denticola* disrupt the tight junctions of the epithelial monolayer, thereby facilitating bacterial penetration into underlying periodontal tissues.^[^ ^90^ ^]^ As particles smaller than 100 nm can pass through the extracellular matrix of host cells,^[^ ^91^, ^92^ ^]^ OMVs, as nanoparticles secreted by gram‐negative bacteria, are more likely to invade epithelial cells and further penetrate the intercellular spaces in the epithelium into deep periodontal tissues than the bacteria themselves. OMVs can carry virulence factors deep into periodontal tissues and act on innate immune cells and connective tissues.^[^ ^91^ ^]^ OMVs from *T. forsythia* increase the levels of IL‐6, IL‐8, and MCP‐1 in periodontal ligament fibroblasts, and these pro‐inflammatory cytokine levels are notably higher than those induced by whole *T. Forsythia*.^[^ ^80^ ^]^ Pro‐inflammatory cytokines can activate immune cells such as macrophages, neutrophils, and lymphocytes, which promote the degradation of connective tissue and resorption of alveolar bone.^[^ ^93^ ^]^ In this study, we found that OMVs from *F. nucleatum* could invade hPDLSCs through endocytosis, among which caveolin‐dependent endocytosis was dominant and triggered the NLRP3 inflammasome and impaired mineralization through the activation of NF‐κB signaling.

Human periodontal ligament stem cells (hPDLSCs), derived from the periodontal ligament (PDL) between the cementum and alveolar bone, play a crucial role in periodontal homeostasis.^[^ ^60^, ^61^, ^94^ ^]^ They have the capacity to form new fibers and cementum and reconstruct alveolar bone to maintain the stability of the internal environment of periodontal tissue when injured or damaged.^[^ ^95^ ^]^ These capacities are attributed to their stem cell characteristics, with potent self‐renewal, secretion mediation, immune regulation and multidirectional differentiation.^[^ ^96^, ^97^ ^]^ When encountering OMVs derived from *F. nucleatum*, hPDLSCs would internalize these OMVs by endocytosis. This was the conventional entry for cells that interacted with OMVs. For *P. gingivalis* OMVs, Furuta et al. showed that OMVs entered human gingival epithelial cells through a lipid raft‐dependent endocytosis.^[^ ^89^ ^]^ For *A. actinomycetemcomitans*, Thay et al. showed that OMVs entered through clathrin‐dependent and lipid raft‐dependent endocytosis.^[^ ^98^ ^]^ For *Helicobacter pylori* OMVs, Olofsson et al. found that OMVs entered gastric epithelial cells via clathrin‐dependent and clathrin‐independent endocytosis.^[^ ^66^ ^]^ In this study, we examined all three pathways, including caveolin‐ and clathrin‐dependent and lipid raft‐endocytosis using immunofluorescence and endocytosis inhibitors (Figure 4a,b; Figure S6, Supporting Information), and observed that all three pathways were involved in endocytosis, especially caveolin‐dependent endocytosis. These results demonstrated that the consistency between the endocytosis of *F. nucleatum* OMVs in hPDLSCs and other results in gingival epithelial cells^[^ ^89^ ^]^ and gastric epithelial cells.^[^ ^66^ ^]^

After internalization of OMVs derived from *F. nucleatum*, hPDLSCs triggered a series of cascade reactions, including cell migration (Figure 4c,d), signaling activation (Figure 7), inflammatory initiatation (Figure 5; Figures S8–S12, Supporting Information) and final cell mineralization (Figure 6). We observed that *F. nucleatum* OMVs did not affect the cell viability or proliferation of hPDLSCs in the short term by the CCK8 assay (Figure S8, Supporting Information), but only influenced cell migration (Figure 4c,d), which was not consistent with the results of inhibition of cell proliferation and even apoptosis of gingival fibroblasts and umbilical vein endothelial cells induced by *P Gingivalis* OMVs.^[^ ^73^, ^79^ ^]^ However, these different results may be due to differences in OMV concentration. However, even at the current concentrations, we found that *F. nucleatum* OMVs could trigger a series of intracellular reactions brought about by the activation of NLRP3 inflammasomes (Figure 5). The NLRP3 inflammasome is a cytosolic signaling complex consisting of NLRP3 (a sensor molecule), ASC, and caspase 1. Microbial infections and endogenous danger signals activate NLRP3, and ASC and caspase 1 are recruited, resulting in the release of the proinflammatory factors IL‐1β and IL‐18.^[^ ^99^ ^]^ Recent reports have confirmed that NLRP3 inflammasome plays an important role in the initiation and deterioration of periodontitis.^[^ ^100^, ^101^ ^]^ In the current study, we showed that *F. nucleatum* OMVs induced a cascade of NLRP3 inflammasomes including ROS and NLRP3 inflammasomes, and the secretion of inflammatory factors, such as TNF‐α, IL‐6, IL‐1β, and IL18 in hPDLSCs, which was similar to other results.^[^ ^78^, ^82^, ^102^, ^103^ ^]^ Pathways including NF‐κB, Wnt/β‐catenin and MAPK have been reported to influence the inflammatory microenvironment and impact mineralization of hPDLSCs.^[^ ^104^ ^]^ Engevik et al. reported that *F. nucleatum* OMVs promoted the secretion of inflammatory factors through the ERK/MAPK and NF‐κB pathways in human colonoid monolayers.^[^ ^43^ ^]^ LPS decreases the osteogenic potential of hPDLSCs by activating the NF‐κB pathway.^[^ ^105^ ^]^ In the current study, we found that *F. nucleatum* OMVs activated NF‐κB signaling and p38/MAPK signaling in hPDLSCs, with NF‐κB signaling showing the most significant change. Blocking NF‐κB signaling could impair OMV‐activated NLRP3 inflammasomes, suggesting that *F. nucleatum* OMVs regulated the NLRP3 inflammasome mainly via NF‐κB signaling. In addition, blocking NF‐κB signaling partially rescued OMV‐impaired mineralization of hPDLSCs, further inferring its regulatory role in hPDLSC function induced by *F. nucleatum* OMVs. These findings suggest the importance of NF‐κB signaling; however, the impact of other pathways, such as MAPK signaling cannot be ignored and warrants further research.

The most direct consequence of inflammation in hPDLSCs triggered by *F. nucleatum* OMVs is impairment of osteogenic differentiation and mineralization. Owing to the crucial role of hPDLSCs in repairing and maintaining periodontal tissue, the impairment in osteogenic differentiation and mineralization seemed to be a key factor in the entire periodontal tissue lesion and alveolar bone loss. We noticed that previous reports often focused on some specific virulence factors, such as LPS and GroEL, which could greatly weaken the osteogenic differentiation and mineralization of periodontal stem cells,^[^ ^72^, ^106^, ^107^ ^]^ and reports also indicated that these virulence factors could trigger cytoplasmic signaling pathways, such as Wnt/β‐catenin, MAPK, and NF‐κB.^[^ ^43^, ^104^, ^105^, ^108^ ^]^ However, none of these reports, like the OMVs in the current study, contained multiple types of virulence factors that were the same as the bacteria themselves, and showed the integrated activation of cytoplasmic signaling as well as the integrated reduction in osteogenic differentiation and mineralization. In the current study, we sequenced and analyzed the proteins in *F. nucleatum* OMVs using protein mass spectrometry and found that *F. nucleatum* OMVs contain a variety of virulence protein components that are correlated with the pathogenicity of periodontitis. Importantly, it's well known that OMVs also contains other components such as polysaccharides, lipids, and nucleic acids, which may also have toxic effects.^[^ ^34^, ^35^ ^]^ Choi et al. found that *P. gingivalis* OMVs contain miRNA‐sized small RNAs (msRNAs), which can be transported to host cells, identify putative immune‐related target genes, and prevent T cells from expressing particular cytokines.^[^ ^109^ ^]^ *P. gingivalis* OMVs contain LPS, which activates the host innate immune response and is associated with both the formation and composition of OMVs.^[^ ^110^ ^]^ However, the specific role of these components in OMVs and the mechanisms involved require further investigation. In this study, we considered OMV‐containing multiple components as a whole to study their periodontitis pathogenic effects on periodontitis, and further research is needed to determine the importance of these individual components in OMV‐induced periodontitis. Based on the results that OMVs could independently cause the occurrence of periodontitis in a rat model (Figures 1 and 2), we emphasized the importance and irreplaceability of OMVs as a pathogenic entity. At the same time, we could also understand that the occurrence and progression of periodontitis may be the result of the combined action of various OMVs carrying many virulence factors derived from a series of periodontal pathogens, including *F. nucleatum*.

## 4 Experimental Section

### Bacteria Culture

*F. nucleatum* (ATCC 25 586) was obtained from Chinese Oral Microbiology Resource Database. *F. nucleatum* was cultured in brain heart infusion (BHI) medium (No.237500, BD Biosciences, NJ, USA) supplemented with 0.005‰ hemin (502 901, J&K Scientific, Beijing, China), 0.001 Phylloquinone (K1) (No.47773, J&K Scientific, Beijing, China), and blood plates (No.024070, Huankai Microbial, Guangdong, China) in an anaerobic atmosphere of 10% CO_2_, 80% N_2_, and 10% H_2_ at 37 °C.

### Isolatoion and Purification of *F. Nucleatum* Outer Membrane Vesicles

The procedures for isolating and purifying *F. nucleatum* outer membrane vesicles (OMVs) were performed according to the previously described methods, with modifications.^[^ ^42^, ^43^ ^]^ Briefly, after *F. nucleatum* cultured anaerobically in 800 ml BHI medium at 37 °C for 2 d, the bacteria were isolated by centrifugation at 10 000 × g for 20 min and the culture supernatant was collected. The supernatant with a 0.45 µm filter (PR05542, Millipore, MA, USA) and further concentrated using an ultrafiltration tube (UFC910096, Millipore, MA, USA) at 5000 rpm for 30 min. The vesicle‐containing supernatant was ultracentrifuged for 2 h at 100 000 × g and 4 °C. Density gradient centrifugation with OptiPrep solution (D1556, Sigma–Aldrich, St. Louis, USA) was used to purify the OMVs. The pelleted OMVs were mixed in 40% OptiPrep solution at the bottom layer of the ultraclear centrifuge tube, followed by 35%, 30%, 25%, 20%, 15% and 10% OptiPrep solution in sequence and ultracentrifuged for 4 h at 100 000 × g and 4 °C. It was obtained ≈1.8 ml OMVs samples (2.2 mg ml^−1^, ≈5.19E+11 particles (OMVs)/ml) from 800 ml BHI media of *F. nucleatum* (≈5E+7 CFU ml^−1^). Sequential fractions were collected, and 10% SDS‐PAGE was used to identify the fractions containing OMVs. Mixing fractions containing OMVs (15%–25%) were pelleted by ultracentrifugation for 2 h at 100 000 × g and 4 °C. The purified OMVs were resuspended in PBS and stored at ‐80 °C for future use.

### Identification of *F. Nucleatum* OMVs

The OMVs samples were added to a copper mesh and negatively dyed with 1% phosphotungstic acid at room temperature for 1–2 min. The morphology were observed and photographed using transmission electron microscopy (TEM).

The prepared OMVs were diluted in PBS at a ratio of 1:10000. The particle size and concentration of the OMVs samples were analyzed by nanoflow cytometry. For OMVs sample the purity evaluation, 10% Triton X‐100 was added to the OMVs preparation. After incubation on ice for 30 min, the particle concentration of the treated samples was analyzed using nanoflow cytometry.

The prepared OMVs were fixed with 2.5% glutaraldehyde for 2 h and then added to cell crawls. Ethanol dehydration was followed by an evaporated gold coating, and the OMV morphology was observed under a scanning electron microscope (SEM).

### Transmission Electron Microscope (TEM)

*F. nucleatum* cultured anaerobically for 48 h, and the bacteria were collected. The samples were prefixed with 3% glutaraldehyde, refixed with 1% osmium tetroxide, dehydrated with a series of acetone, infiltrated, and embedded with Epox812. Ultra‐thin slices were prepared and stained with uranium acetate and lead citrate. Micrographs of the sections were acquired using a transmission electron microscope (JEM‐1400FLASH).

### Protein Mass Spectrometry (MS)

The prepared OMVs and *F. nucleatum* bacterial samples were sent for LC/MS/MS analysis at Shanghai Ease Biotechnology Co., Ltd. Briefly, total proteins from OMV samples and *F. nucleatum* bacteria samples were extracted, and their concentrations were determined. Next, 100 µg of total protein was digested, and the digested products were washed with 50 mM ammonium bicarbonate, acidified with trifluoroacetic acid, and oscillated, centrifuged to remove sodium deoxycholate, and desalted using a C18 desalting column. To prepare desalted samples for liquid chromatography‐tandem mass spectrometry using a Thermo Q Exactive HF mass spectrometer, the samples were first lyophilized in a freeze dryer. After lyophilization, samples were dissolved in 0.1% formic acid. The mass spectrometry detection of RAW data was performed using UniProt database. The expanded prediction of bacterial protein subcellular localization was obtained from PSORTb version 3.0 using DAVID and InterPro to classify the GO functional annotation and functional enrichment analysis.

### Rat Periodontitis Model

A ligation‐induced rat periodontitis model was established as previously described.^[^ ^33^, ^45^, ^46^ ^]^ All the animal experimental protocols were approved by the Institutional Animal Care and Use Committee (WCHSIRB‐CT‐2022‐127). Five‐week‐old male SD rats were obtained from Chengdu Dashuo Animal Center. For antibiotic treatment, the rats were fed drinking water containing 0.008% sulfamethoxazole and 0.016% trimethoprim for one week for synchronization and then replaced with normal drinking water. After antibiotic treatment for three days, the SD rats were randomly assigned into four groups (non‐ligation + PBS, ligation + PBS, ligation + *F. nucleatum* and ligation + *F. nucleatum* OMVs groups, n = 6). After anesthetization with inhaling isoflurane, a 5‐0 silk ligature was ligated around both sides of the maxillary second molars of the rats. Check if the silk thread falls off every two days. After ligation for two days, the rats were injected separately with PBS, *F. nucleatum* (100 µl, 1×10^9^ CFU ml^−1^), and *F. nucleatum* OMVs (20 µl, 1 µg µl^−1^) into the mesial and distal periodontal tissues of the maxillary second molar three times per week. For the experiment on *F. nucleatum* OMV‐induced periodontitis, the SD rats were randomly assigned to either the control group or *F. nucleatum* OMVs group (n = 6), and the rats were injected separately with PBS and *F. nucleatum* OMVs (20 µl, 1 µg µl^−1^, ≈2.36E+9 particles (OMVs)) into mesial and distal periodontal tissues of the maxillary second molar 3 times per week. On the 28th day of administration, all rats were cervical dislocated to euthanized and their maxillae were retrieved and fixed with 4% paraformaldehyde (PFA).

### Micro‐Computed Tomography (µ‐CT)

After fixing in 4% PFA overnight, PFA was washed off with water and dehydrated the maxilla samples in 70% ethanol. The samples were then scanned using a Micro‐CT Scanner (Scanco). The scanning parameters included the tube potential (70 kVp), intensity (200 µA), exposure time (300 ms), and resolution (10 µm). 3D reconstruction of mineralized tissues was performed using SCANCO Medical Evaluation and SCANCO Medical Visualizer software. The area from the cementoenamel junction to the alveolar bone crest of the maxillary second molar was analyzed.

### Hematoxylin and Eosin (HE) and Masson Staining

After immersion in xylene for dewaxing and immersion in graded alcohol for rehydration, the maxilla sections were stained with HE and Masson's trichrome stain to observe changes in periodontal tissues. Staining was performed according to the manufacturer's instructions (G1340, Solarbio). Images were obtained using a microscope slide scanner (Olympus SLIDEVIEW VS200, Japan).

### Tartrate Acid Phosphatase (Trap) Staining

Paraffin‐embedded sections were immersed in xylene for dewaxing and in graded alcohol for rehydration. The slices were then stained using the Trap stain kit (FUJIFILM Wako). Images were obtained using a microscope slide scanner. Osteoclasts with Trap positivity in the alveolar bone around the maxillary second molar were analyzed using Image J software (v2.9.0, NIH, Bethesda, MD, USA).

### Immunohistochemistry (IHC) Staining Analysis

After deparaffinization and hydration, the sections were immersed in a citrate antigen repair solution at 100 °C for 18 min. Then, the sections received a 3% H_2_O_2_ treatment for 30 min before being sealed with 5% goat serum for 1 h. Afterward, the specimens were respectively treated with relevant primary antibodies: anti‐NLRP3 (381 207, Zen‐Bio, Chengdu, China, 1:200 dilution), anti‐IL1β (RB20040UC, Zen‐Bio, Chengdu, China, 1:200 dilution), anti‐IL18 (10663‐1‐AP, Proteintech, Wuhan Sanying, Wuhan, China, 1:200 dilution), anti‐Cathepsin K (11239‐1‐AP, Proteintech, 1:200 dilution), anti‐DCST1 (orb2242, Biorbyt, Cambridge, UK, 1:200 dilution), anti‐IL6 (No.500286, Zen‐Bio, 1:200 dilution), anti‐TNFα (No.346654, Zen‐Bio, 1:200 dilution) and anti‐Collagen I (No.343277, Zen‐Bio, 1:200 dilution) at 4 °C for 12 h. Afterward, the sections were treated with IgG secondary antibodies (1:200, PK‐2200, Vectorlabs, CA, USA) for 2 h at room temperature, followed by visualizing the positive immune signal using DAB solution kit (SK‐4100, Vectorlabs, CA, USA). Images were acquired using a microscope slide scanner. Quantitative analysis of positive immune signals was performed using the Image J software.

### Tissue Immunofluorescence

Maxilla sections were immersed in xylene for dewaxing and in graded alcohol for rehydration, followed by heat‐induced epitope retrieval. Then, the sections were treated with 3% H_2_O_2_ for 30 min and then 5% goat serum for 1 h. Afterward, the sections were treated with anti‐DCST1, anti‐CD11c (ab11029, Abcam, 1:200), anti‐CD3 (ab5690, Abcam, 1:200), anti‐Cathepsin K (1:200), anti‐F4/80 (ab100790, Abcam, 1:200), anti‐MMP9 (ab38898, Abcam, 1:200), anti‐NLRP3 (1:200), anti‐IL1β (1:200), anti‐IL18 (1:200), anti‐IL6 (1:200), anti‐TNFα (1:200), anti‐Runx2 (No.860139, Zen‐Bio, 1:200), anti‐MMP2 (ab97779, Abcam, 1:200), anti‐ALP (R23427, Zen‐Bio, 1:200) and anti‐OSX (ER1914‐47, Huabio, 1:200) at 4 °C for 12 h. Next, the specimens were incubated with fluorescein‐conjugated anti‐rabbit and anti‐mouse IgG secondary antibody (Alexa Fluor 488, ab150073 and ab150077, Abcam, 1:200) overnight at 4 °C. Nuclei were staining with DAPI (D9542, Sigma, MO, USA). Fluorescence images of the tissues were obtained using a microscope slide scanner. Quantitative analysis of the fluorescence intensity was performed using Image J software.

### Cell Culture

The protocol was approved by the Human Research Ethics Committee of the West China Hospital of Stomatology, Sichuan University (WCHSIRB‐2021‐004, Chengdu, China) and informed written consent was obtained from all human patients involved in this research. hPDLSCs were harvested as previously described.^[^ ^5^ ^]^ In brief, the periodontal ligaments of the middle third of the root were scraped from extracted healthy human premolars and third molars for orthodontic therapy (aged 12–24 years). The PDL samples were treated with 0.3% collagenase I (17 100 017, Gibco, MA, USA) at 37 °C for 30 min. After centrifugation at 1000 rpm for 5 min to remove the collagenase I solution, the PDL tissue was cultured in culture dishes with α‐MEM media containing 1% penicillin‐streptomycin (Hyclone) and 10% fetal bovine serum (FBS) at 37 °C in an incubator with 5% CO_2_. Subculture was performed when the cells reached ≈80% confluence and hPDLSCs (passages 3–5) were used in the present study.

### Cell Counting Kit‐8 (CCK‐8) Assay

hPDLSCs were seeded into 96‐well plates for 24 h, and different concentrations of *F. nucleatum* OMVs (0, 0.1, 0.5, 1, 5, 10, 25, and 50 µg ml^−1^), dynasore (0, 20, 50, 80, and 100 µM), methyl‐β‐cyclodextrin (0, 0.5, 1, 5, and 8 mM), and chlorpromazine (0, 5, 15, 30, and 50 µg ml^−1^) were used to stimulate the cells. At 0.5, 1, 3, and 5 days after treatment with *F. nucleatum* OMVs, 10% CCK‐8 solution was added to the wells and the absorbance was measured at 450 nm after 1 h.

### Wound‐Healing Assay

The hPDLSCs were seeded into 96‐well plates. After the cells reached 100% confluence, hPDLSCs were cultured in 2% FBS α‐MEM media for 12 h. A sterile tip was used to apply a linear scratch in the middle of the container cell well, which was then washed with PBS. Subsequently, the cells were grown in 2% FBS α‐MEM medium with 0, 15, and 30 µg ml^−1^ of *F. nucleatum* OMVs for 40 h. hPDLSCs migration was captured at 0, 16, and 40 h after scratching and *F. nucleatum* OMVs stimulation using phase‐contrast microscopy. ImageJ software was used to analyze changes in the mobility ratio (migrated cell area/scraped region).

### Exosome Labelling and Internalization

An amount of 8 µM Dil (D282, Invitrogen) was incubated with *F. nucleatum* OMVs for 30 min to label the *F. nucleatum* OMVs in accordance with a previous study.^[^ ^42^ ^]^ hPDLSCs were seeded onto glass bottom dishes and cultured in 10% FBS α‐MEM media for 12 h. For time‐dependent internalization studies, the hPDLSCs were treated with 30 µg ml^−1^ Dil‐labeled *F. nucleatum* OMVs after 0, 1, 2, and 4 h. To identify the entry pathway of *F. nucleatum* OMVs into hPDLSCs, hPDLSCs were pretreated with various endocytosis inhibitors, including 80 µM dynasore (HY‐15304, MCE, NJ, USA), 5 mM methyl‐β‐cyclodextrin (MβCD, HY‐101461, MCE, NJ, USA), and 15 µg ml^−1^ chlorpromazine (HY‐12708, MCE, NJ, USA) for 1 h. hPDLSCs without inhibitor treatment were used as the control group. Then, Dil‐labeled *F. nucleatum* OMVs were stimulated with hPDLSCs and co‐cultured for 1 h. Subsequently, immunofluorescence staining was performed in the dark. The cells were fixed with 4% PFA and sealed with 5% BSA for 1 h. Cells were then incubated with FITC‐phalloidin (A12379, Invitrogen, CA, USA) overnight at 4 °C to label the cytoskeleton and stained with DAPI for 10 min to visualize the cell nuclei. CLSM (FV3000, Olympus, Japan) was used to obtained fluorescence images of hPDLSCs. Quantitative analysis of the fluorescence intensity was performed using ImageJ software.

For the colocalization of *F. nucleatum* OMVs with caveolin and clathrin, Dil‐labeled OMVs were added to hPDLSCs, incubated for 1 h, and processed as described above. After sealing with 5% BSA, the samples were immersed in primary antibodies: anti‐clathrin (381 847, Zen‐Bio, 1:200) and anti‐caveolin (R22762, Zen‐Bio, 1:200) for 12 h at 4 °C. Next, samples were incubated with fluorescein‐conjugated anti‐rabbit secondary antibody (ab150075, Abcam, 1:200) for 2 h, followed by incubation with FITC‐phalloidin to label the cytoskeleton and DAPI to visualize cell nuclei. Fluorescence images were obtained using CLSM.

### Measurement of Intracellular f Reactive Oxygen Species (ROS)

hPDLSCs were seeded into 6‐well plates (for flow cytometry analysis) and glass‐bottom dishes (for IF analysis). Intracellular ROS levels were measured using a DCFH‐DA probe (S0033S, Beyotime, Shanghai, China). The cells were grown in 10% FBS α‐MEM media in the presence or absence of 30 µg ml^−1^ *F. nucleatum* OMVs for 6 h, ROS staining solution was added to the wells, and the cells were incubated for 20 min. Next, the cells were rinsed three times with serum‐free medium to remove the remaining ROS. Flow cytometry and CLSM were used to detect intracellular ROS levels following the manufacturer's instructions.

### Immunofluorescence Analysis and Confocal Laser Scanning Microscopy (CLSM)

For IF analysis, hPDLSCs were seeded onto glass‐bottom dishes and treated with or without *F. nucleatum* OMVs (30 µg ml^−1^) for 6 or 24 h. After rinsing three times with PBS, hPDLSCs were fixed in 4% cold PFA. The samples were then sealed with 5% BSA for 1 h after permeation with 0.25% Triton X‐100 solution for 10 min. The hPDLSCs were then incubated with anti‐NLRP3 (381 207, Zen‐Bio, 1:200) and anti‐phospho‐NF‐κB p65 (No.3033, Cell Signaling Technology, MA, USA, 1:200) for 12 h at 4 °C. The hPDLSCs were then incubated with fluorescein‐conjugated anti‐rabbit secondary antibody for 2 h, followed by incubation with FITC‐phalloidin to label the cytoskeleton and DAPI to visualize cell nuclei. CLSM was used to obtain fluorescence images of hPDLSCs. Quantitative analysis of the fluorescence intensity was performed using ImageJ software.

### Western Blotting

To detect changes in inflammatory factors and signal pathway proteins in *F. nucleatum* OMV‐simulated hPDLSCs, hPDLSCs were seeded into 6‐well plates and cultured in 10% FBS α‐MEM medium. The cells were then treated with or without *F. nucleatum* OMVs (30 µg ml) for 0.5, 1, 4, 6, 12, or 24 h at 37 °C. To determine changes in proteins related to osteogenic differentiation in *F. nucleatum* OMV‐simulated hPDLSCs, hPDLSCs were seeded into the 6‐well plates and cultured in mineralization medium (ascorbic acid (0.05 M, PHR1008, Sigma), dexamethasone (100 nM, D8893, Sigma) and β‐glycerophosphate (8 mM, G9891, Sigma)). The cells were treated with or without *F. nucleatum* OMVs (30 µg ml) for 3, 7, and 14 days at 37 °C. For the signal pathway inhibition experiments, hPDLSCs were pretreated with 5 µM SC75741 for 1 h and then treated with or without OMVs (30 µg ml^−1^). Cell samples were harvested at 6 h and 1 d (for western blotting).

The cells were lysed using a radio immunoprecipitation assay (RIPA, 68 117 726, Biosharp, Guangdong, China) containing a protease inhibitor after rinsing with PBS. Protein concentrations in the cell lysate samples were measured using a BCA assay. Protein samples were denatured for 5 min at 100 °C after mixing with 1 × loading buffer. After electrophoresis, the proteins were transferred to PVDF membranes. Next, the membranes were sealed with 5% skim milk and then incubated respectively with primary antibodies at 4 °C for 12 h: anti‐β‐actin (T200068‐8F10, ZEN BIO, 1:1000), anti‐MMP2 (1:1000), anti‐MMP9 (1:1000), anti‐NLRP3 (1:1000), anti‐IL‐1β (1:1000), anti‐IL‐18 (1:1000), anti‐IL‐6(1:1000), anti‐TNF‐α, anti‐Phospho‐NF‐κB p65 (1:1000), anti‐Phospho‐JNK1 (No.381100, ZEN BIO, 1:1000), anti‐Phospho‐p38‐MAPK (No.310091, ZEN BIO, 1:1000), anti‐Phospho‐ERK1 + Phospho‐ERK2 (ab201015, Abcam, 1:1000), anti‐phospho‐β‐catenin (R381527, ZEN BIO, 1:1000), anti‐JNK (No.380556, ZEN BIO, 1:1000), anti‐NF‐κB p65 (No.250060, ZEN BIO, 1:1000), anti‐p38 (No.340697, ZEN BIO, 1:1000), anti‐ERK1 + ERK2 (ab184699, Abcam, 1:1000), anti‐β‐catenin (R23616, ZEN BIO, 1:1000), anti‐Runx2 (1:1000), anti‐Collagen I (1:1000), anti‐ALP (1:1000) and anti‐Osx (1:1000). The membranes were then detected using chemiluminescence reagents (P90719, Millipore, MA, USA) after incubation with the secondary antibody for 2 h at room temperature. The intensity of the membranes was analyzed using the ImageJ software.

### Quantitative Real‐Time Polymerase Chain Reaction (qPCR)

The hPDLSCs were seeded into 6‐well plates and cultured in 10% FBS α‐MEM medium. The cells were then treated with or without *F. nucleatum* OMVs (30 µg ml^−1^) for 12 and 24 h at 37 °C. A total RNA fast isolation Kit (RP1202, Bio Teke, Beijing, China) was used to extract RNA of cells, and the detailed protocols were following the instructions. After quantification with a Nanodrop spectrophotometer, 1 µg RNA was transformed into cDNA using a reverse transcription kit (K1621, Thermo Fisher Scientific, MA, USA). Subsequently, cDNA, primer pairs, nuclease‐free water and TB Green (RR420A, TAKARA, Osaka, Japan) were mixed, and then subjected to qRT‐PCR on an iCycler. The ΔΔCt method, with GAPDH as an internal control, was used to analyze the expression of related genes. Table S1 (Supporting Information) lists the primer sequences for the genes detected in this study.

### Gelatin Zymography

The hPDLSCs were seeded into 6‐well plates and grown in 10% FBS α‐MEM medium. Various concentrations of *F. nucleatum* OMVs (0, 5, 15, and 30 µg ml^−1^) were used to induce the cells. The cell culture supernatant was collected at 12, 24, 48, and 72 h. The protein concentrations of the supernatant samples were determined using a BCA assay before the samples were combined with the loading buffer. Proteins were separated by gelatin SDS‐PAGE. The gels were then treated with proteolysis buffer overnight at 37 °C after being rinsing three times with 2.5% Triton X‐100 solution. After rinsing with 2.5% Triton X‐100 solution for 30 min, the gels were stained with Coomassie blue solution for 2 h and gently decolorized with Destain buffer. The area where gelatinase was located was shown as a white band. Quantitative analysis of band densities was performed using ImageJ software.

### Alkaline Phosphatase Staining

The hPDLSCs were seeded into 48‐well plates and cultured in mineralization medium. After reaching 80% confluence, the cells were stimulated with various concentrations of *F. nucleatum* OMVs (0, 5, 15, and 30 µg ml^−1^) for 7 days. For signal pathway inhibition experiments, hPDLSCs were pretreated with 5 µM SC75741 for 1 h and then stimulated with or without OMVs (30 µg ml^−1^) for 7 days. The alkaline phosphatase (ALP) activity of hPDLSCs was examined using a BCIP/NBT Alkaline Phosphatase Color Development Kit (C3206, Beyotime, Shanghai, China). Briefly, the samples were fixed in 4% PFA and stained in the dark with BCIP/NBT dye solution for 30 min. The samples were then rinsed with PBS to remove any remaining dye. ALP staining images were observed using a light microscope (Olympus IX71, Japan).

### Alizarin Red Staining

The hPDLSCs were seeded into 24‐well plates and cultured in osteogenic differentiation medium. After reaching 80% confluence, the cells were stimulated with various concentrations of *F. nucleatum* OMVs (0, 5, 15, and 30 µg ml^−1^) for 21 days. For signal pathway inhibition experiments, hPDLSCs were pretreated with 5 µM SC75741 for 1 h and then stimulated with or without OMVs (30 µg ml^−1^) for 21 days. After fixation with 4% PFA and rinsing with ddH_2_O, the cells were stained with 1% Alizarin Red staining solution (G1452, Solabio, Beijing, China) for 1 h and then rinsed with ddH_2_O. Mineralized nodule images were obtained using a light microscope.

### RNA Sequencing

The hPDLSCs were seeded onto the 6‐well. After reaching 100% confluence, hPDLSCs were treated with or without *F. nucleatum* OMVs (30 µg ml^−1^) for 2d at 37 °C. TRIzol reagent (15596018CN, Thermo Fisher Scientific, MA, USA) was used to collect the cell samples. The samples were sent to Shanghai Lifegenes Biotechnology Co., Ltd for RNA sequencing analysis. A Bioanalyzer 2100 system (RNA Nano 6000, Agilent Technologies) was used to check RNA quality before sequencing. The data were analyzed using HTSeqv0.6.1 for gene reading. Gene expression was described in the format of million fragments per thousand bases (FPKM). Using GO and KEGG enrichment methods to analyze the differentially expressed genes.

### Statistical Analysis

During data preprocessing, the outliers in the data were preserved as much as possible and incorporated into the data evaluation and statistics. The results of at least three independent experiments (n ≥ 3) are presented as mean ± SD and were drawn using GraphPad Prism. Statistical differences were analyzed using two‐tailed Student's t‐test to evaluate the variability between the two sets, and one‐way analysis of variance (ANOVA) was used to evaluate the variability between multiple sets. Post hoc analysis employed Fisher's protected least significant differences (PLSD), and the threshold of significance levels was set at 0.05 in each analysis.

## Conflict of Interest

The authors declare no conflict of interest.

## Author Contributions

L.Z., D.Z., and C.L. contributed equally to this work. J.X., X.L., and C.L. performed conceptualization; L.Z., D.Z., C.L., M.D., H.Z., Y.L., and Z.X. performed investigation and Data acquisition/curation; L.Z., D.Z., C.L., M.D., Y.T., H.Z., Y.L., H.C., and Z.X. performed formal analysis; B.T., Y.C., X.N., and Y.Z. provided the support for methodology and software; J.X., X.L., and C.L. performed funding acquisition and experimental supervision; L.Z., and M.H. wrote the original draft; J.X., X.L., and C.L. performed writing, reviewing, and editing. All authors have given approval to the final version of the manuscript.

## Supporting information



Supporting Information

## Data Availability

The data that support the findings of this study are available from the corresponding author upon reasonable request.
