# Supplementary material for: Outer Membrane Vesicles Derived From Fusobacterium nucleatum Trigger Periodontitis Through Host Overimmunity
Source: Adv Sci (Weinh). 2024 Oct 30;11(47):2400882. doi: 10.1002/advs.202400882 (PMC11653712; doi:10.1002/advs.202400882)
Supplement: Supplementary file 1 — Supporting Information [file ADVS-11-2400882-s001.doc]

***Supporting Information***

**Outer Membrane Vesicles Derived from Fusobacterium nucleatum Trigger Periodontitis through Host Overimmunity**

Li Zhang^1#^, Demao Zhang^1,2#^, Chengcheng Liu^1#^, Boyu Tang^1^, Yujia Cui^1^, Daimo Guo^1^, Mengmeng Duan^1^, Ying Tu^1^, Huiling Zheng^1^, Xinjie Ning^1^, Yang Liu^1^, Haoran Chen^1^, Minglei Huang^1^, Zhixing Niu^1^, Yanfang Zhao^3^, Xiaoheng Liu^2*^, Jing Xie^1*^

**1. Supplementary Figures**

**Figure S1**


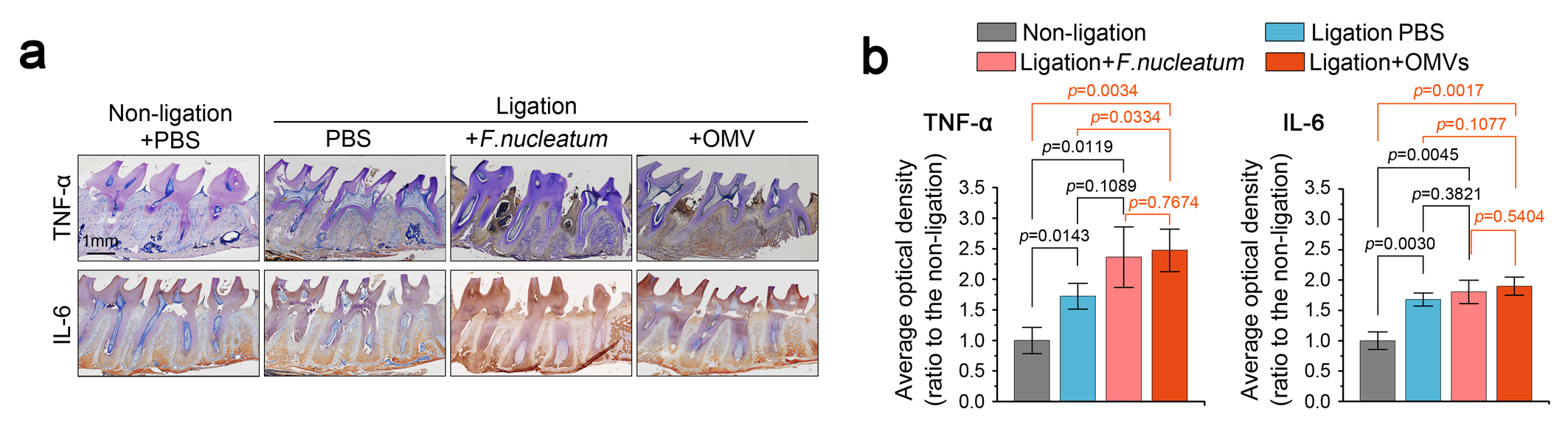


**Figure S1. Immunohistochemical analysis showing the changes of TNF-α and IL-6 in the periodontal tissue of periodontitis induced by *F. nucleatum* and *F. nucleatum* OMVs.**

**(a)** Immunohistochemical images showing the increased expression of TNF-α and IL-6 in the periodontal tissue of periodontitis induced by *F. nucleatum* and *F. nucleatum* OMVs. The images were chosen based on 6 independent experiments (n = 6).

**(b)** Quantitative analysis showing the increase of TNF-α and IL-6 in (a). The data were analyzed based on six independent experiments (n = 6) and presented as mean ± SD.

**Figure S2**


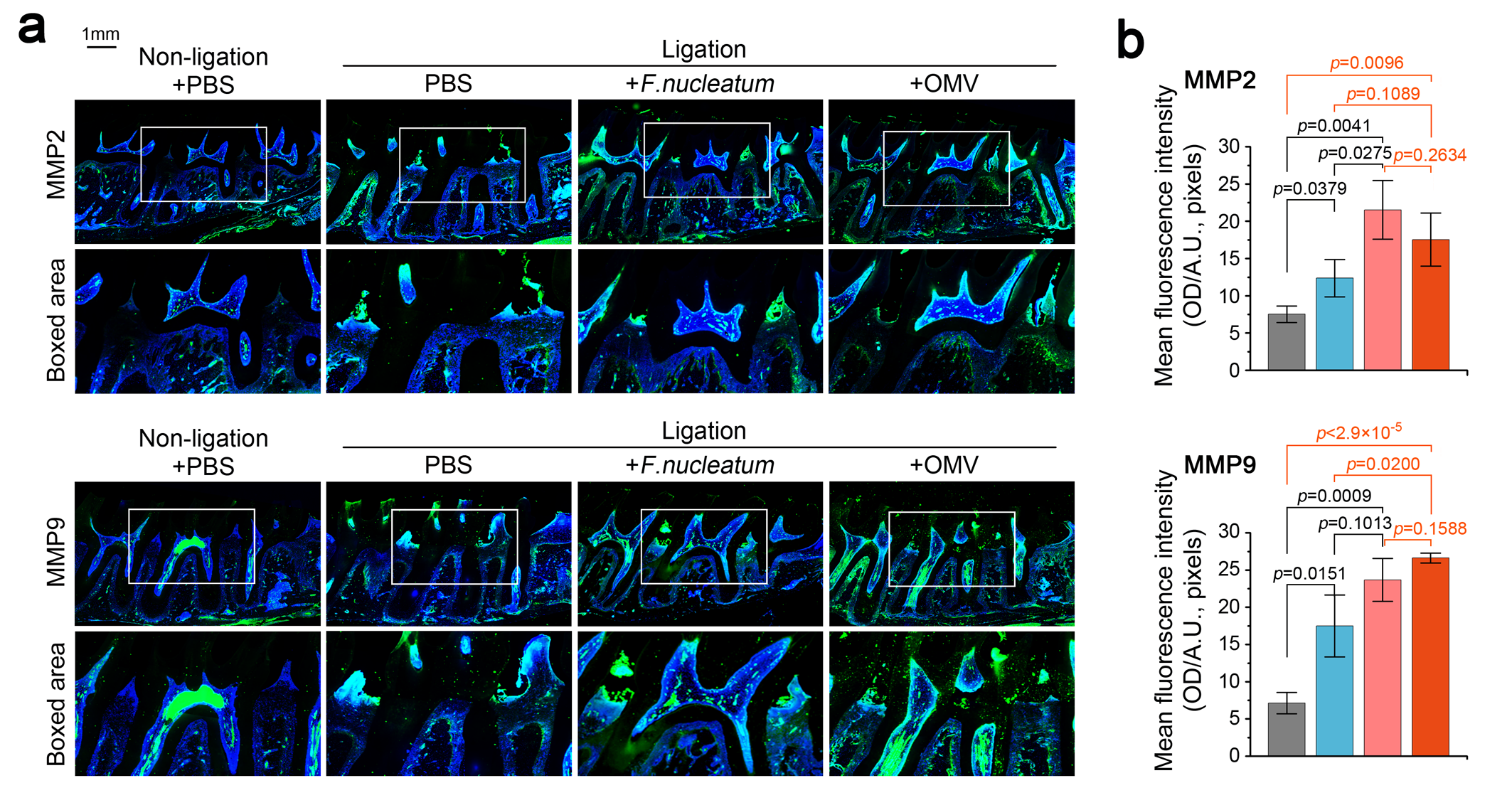


**Figure S2. Immunofluorescent analysis showing the changes of MMP2 and 9 in the periodontal tissue of periodontitis induced by *F. nucleatum* and *F. nucleatum* OMVs.**

**(a)** Immunofluorescent images showing the increase of MMP2 and 9 in the periodontal tissue of periodontitis induced by *F. nucleatum* and *F. nucleatum* OMVs. Images were chosen based on six independent experiments (n = 6).

**(b)** Quantitative analysis based on fluorescence optical density showing the increase of MMP2 and 9 in (a). The data were chosen based on six independent experiments (n = 6) and presented as mean ± SD.

**Figure S3**


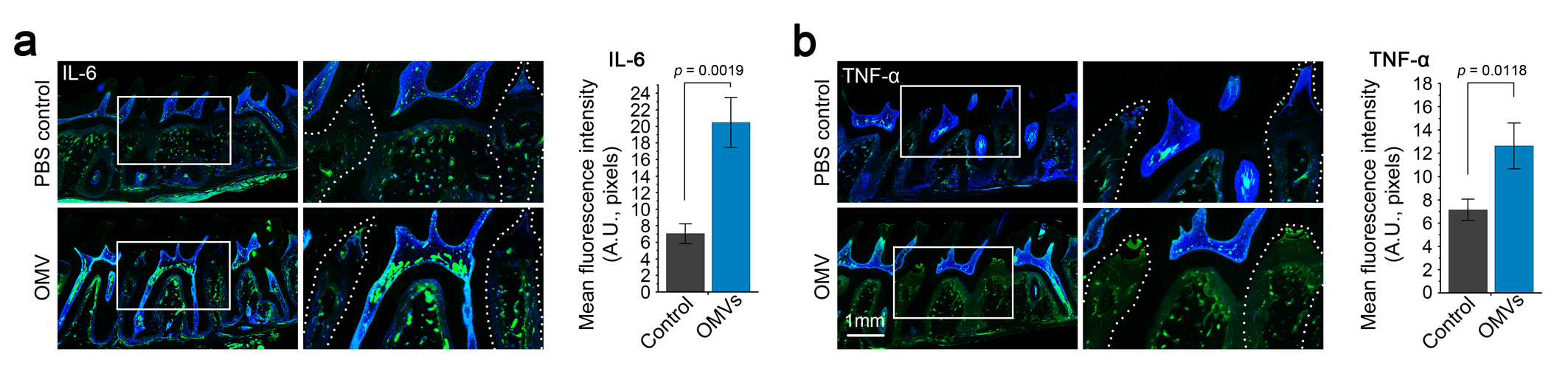


**Figure S3.** **Immunofluorescent analysis showing the changes of IL-6 and TNF-α in the normal periodontal tissue induced by *F. nucleatum* OMVs.**

**(a)** Immunofluorescent images showing the increased expression of IL-6 in the periodontal tissue induced by *F. nucleatum* OMVs. Images were chosen based on six independent experiments (n = 6), and histogram is presented as mean ± SD.

**(b)** Immunofluorescent images showing the increased expression of TNF-α in the periodontal tissue induced by *F. nucleatum* OMVs. Images were chosen based on six independent experiments (n = 6), and histogram is presented as mean ± SD.

**Figure S4**


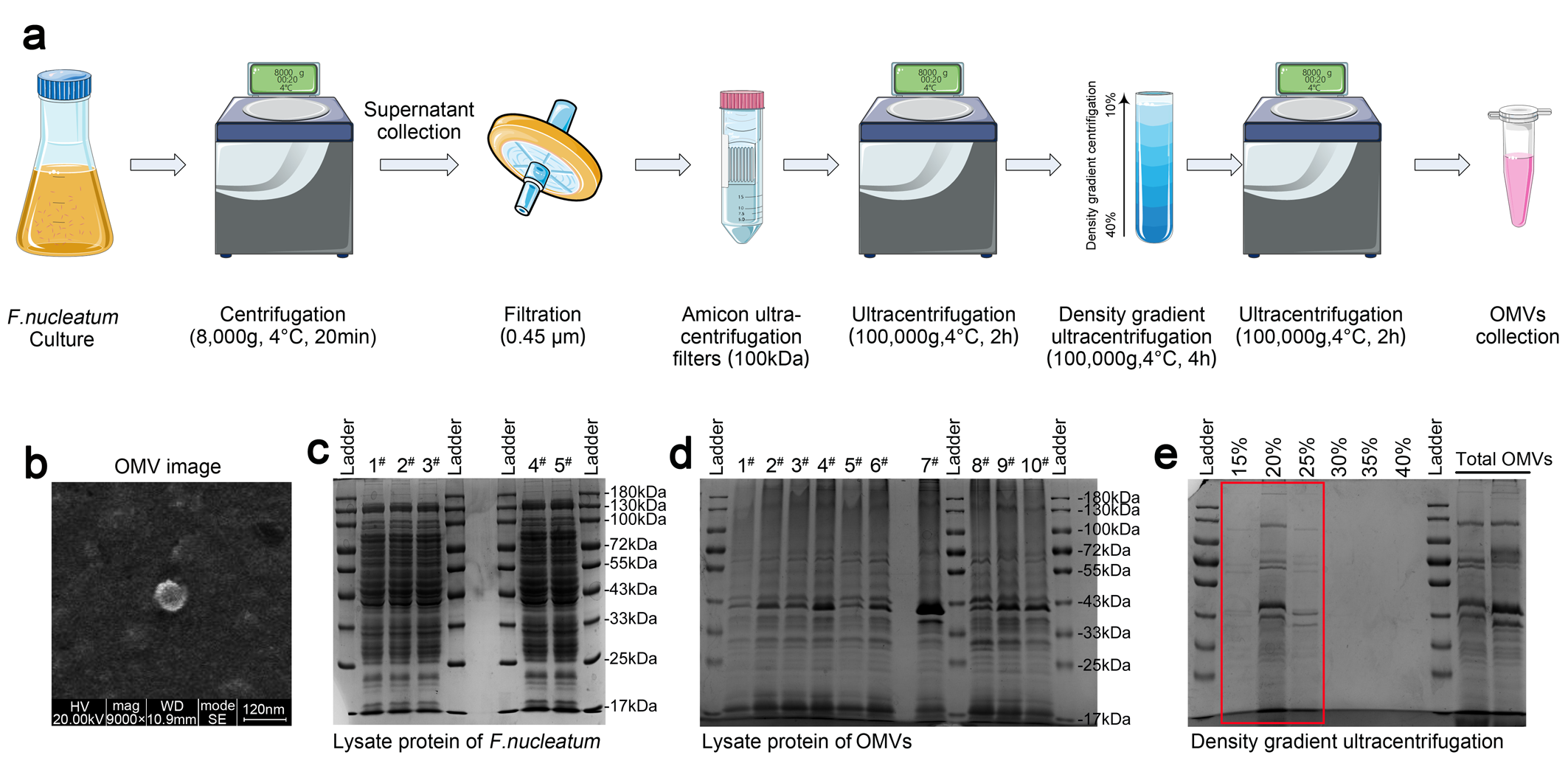


**Figure S4. The isolation process of *F. nucleatum*** OMVs.

**(a)** The diagrammatic sketch showing the total isolation and purification process by through ultrafiltration and ultracentrifugation.

**(b)** Representative SEM images showing the size of OMVs isolated from *F. nucleatum*. Images were chosen based on three independent experiments (n = 3).

**(c)** The total protein profile of *F. nucleatum* by SDS-PAGE electrophoresis. Sample 1-5# were from independent cultures. Images were chosen based on three independent experiments (n = 3).

**(d)** The total protein profile of *F. nucleatum* OMVs by SDS-PAGE electrophoresis. Sample 1-10# were from independent cultures. Images were chosen based on three independent experiments (n = 3).

**(e)** The total protein profile of *F. nucleatum* OMVs through density gradient ultracentrifugation by SDS-PAGE electrophoresis. Images were chosen based on three independent experiments (n = 3).

**Figure S5**


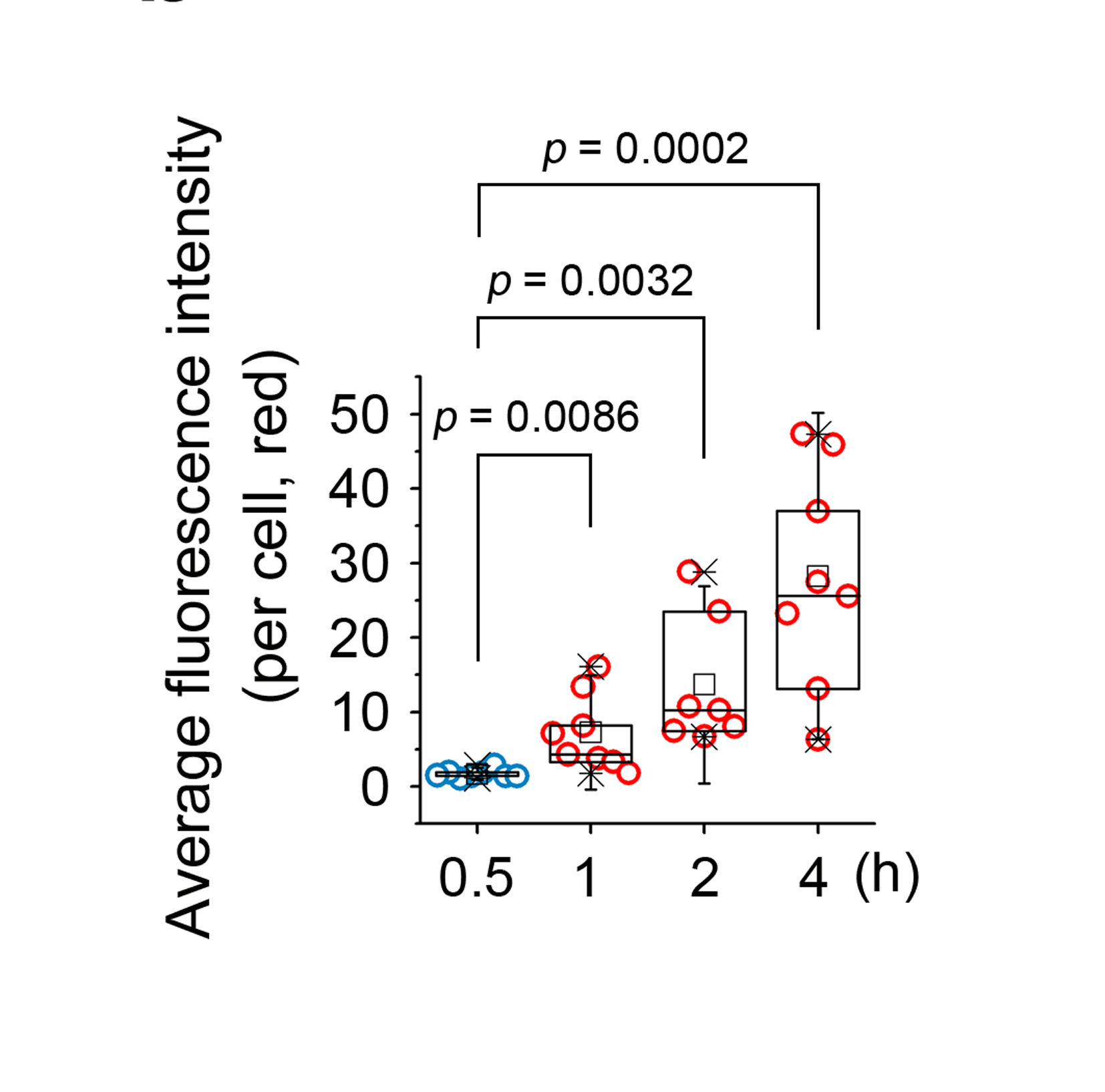


**Figure S5. Quantification of OMVs entering into the cytoplasm of hPDLSCs (per cell) by fluorescent quantification.** Quantitative analysis was based on eight independent experiments (n = 8), and Data are presented as boxes (from 25% to 50 to 75%) and whiskers (standard deviation, SD). This data was referred to Figure 4a.

**Figure S6**


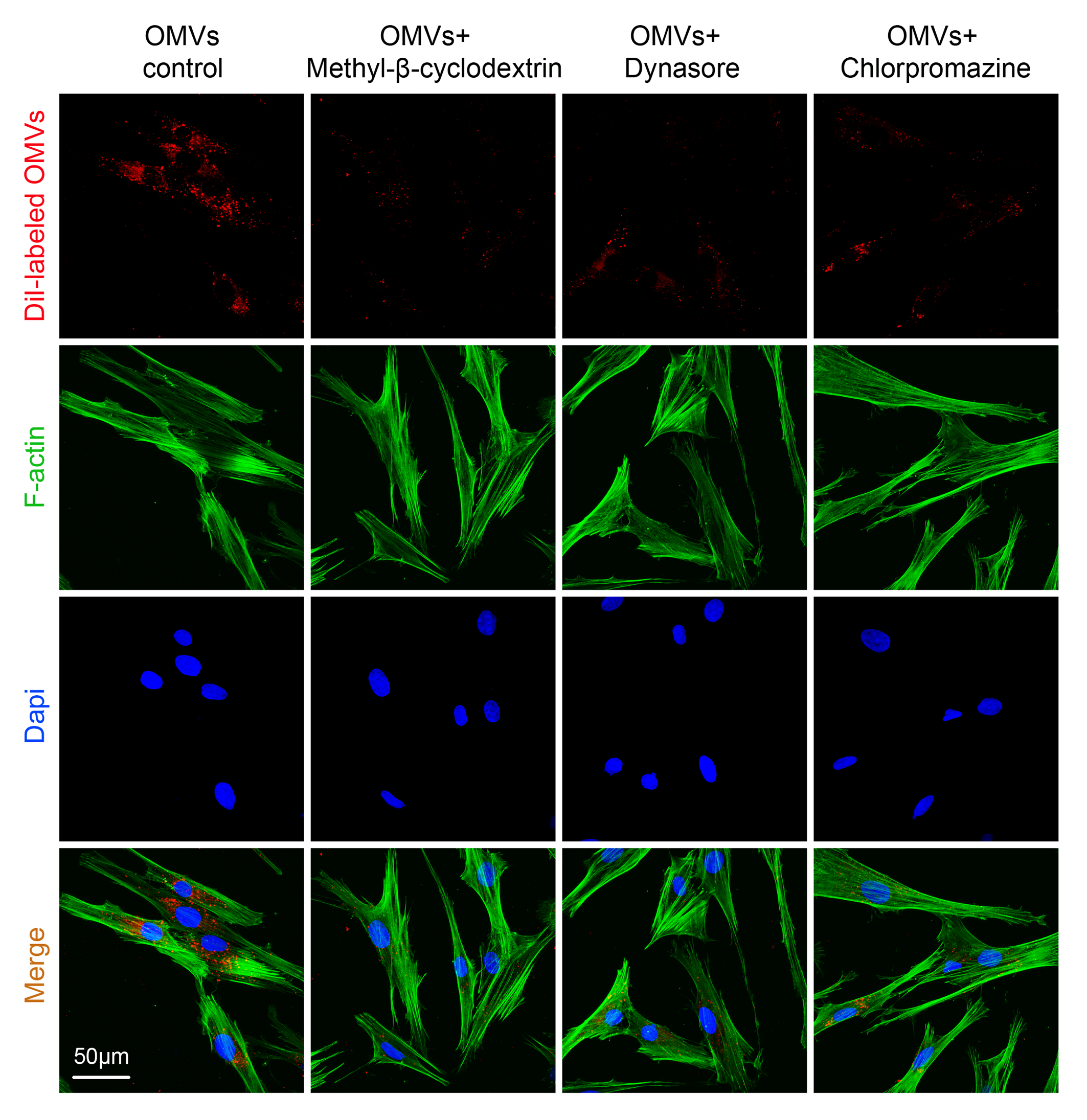


**Figure S6. Inhibitory experiments showing that the OMVs entered the hPDLSCs through endocytosis, especially caveolin-dependent endocytosis.** Dil-labeled OMVs were cocultured for 4 h with hPDLSCs that had been pretreated (1 h) with or without inhibitors of endocytosis including methyl-β-cyclodextrin (5 mM), dynasore (80 μM) or chlorpromazine (15 μg/ml). IF staining by CLSM shows the number of OMVs entering the hPDLSCs sharply decreased with endocytosis inhibitors pretreatment. DiI-labeled OMVs, red; F-actin, green; Nucleus, blue.

**Figure S7**


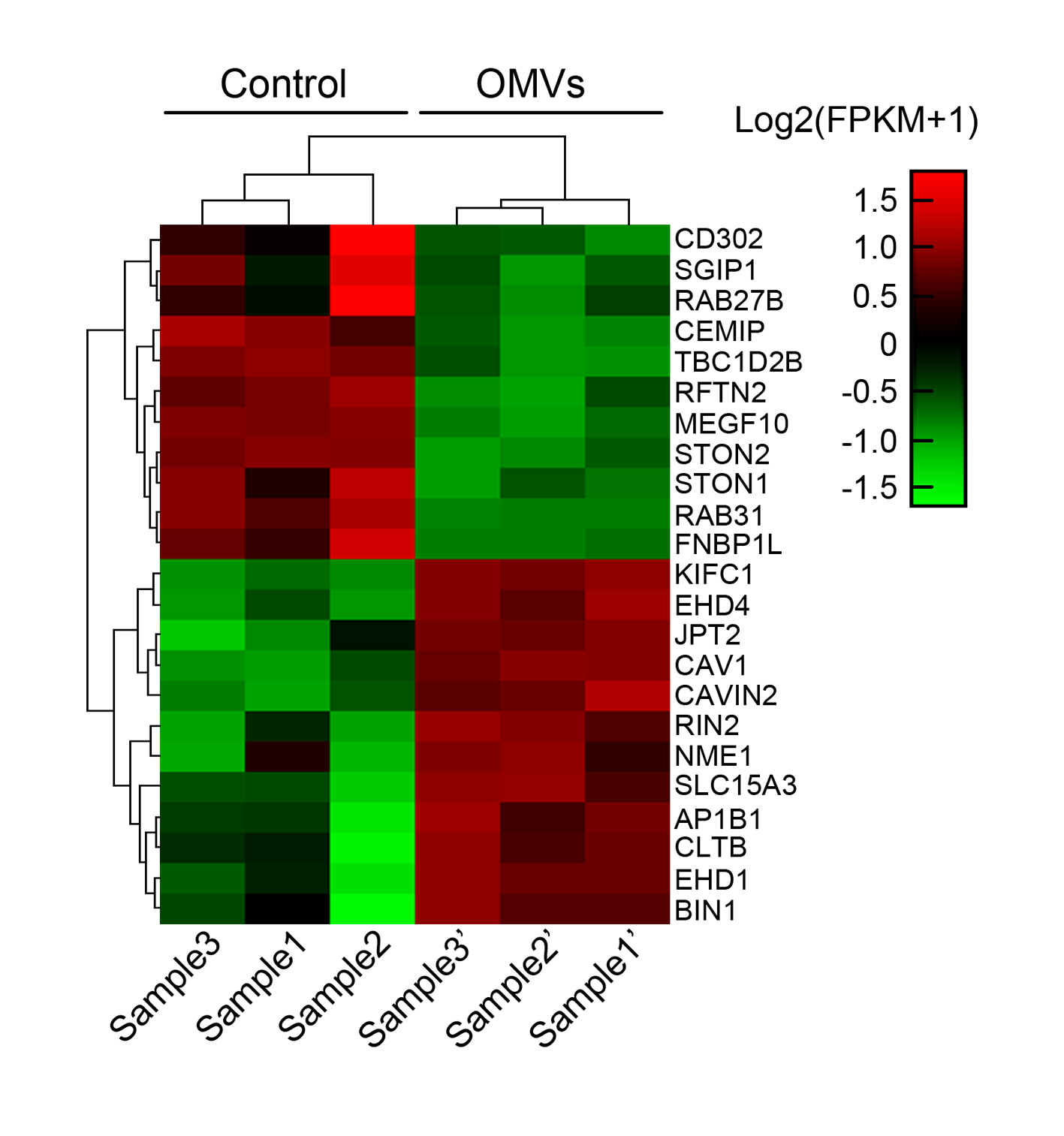


**Figure S7. Pheatmap showing all changed genes related to the endocytosis.** The gene expression of caveolin1 (CAV1) showed the importance of caveolin-dependent endocytosis of hPDLSCs by OMVs. Cell samples 1&1’, 2&2’ and 3&3’ were isolated from the same donators, respectively.

**Figure S8**


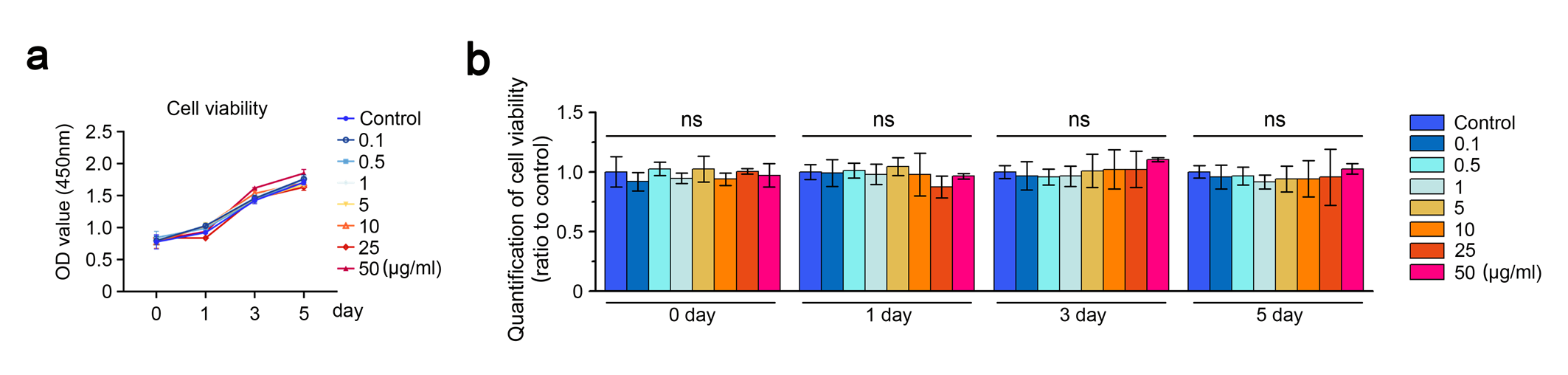


**Figure S8. Cell viability of hPDLSCs induced by OMVs by CCK8 assay.**

**(a)** CCK-8 assay showed that different concentrations（0, 0.1, 0.5, 1, 5, 10, 25 and 50 μg/ml）of *F. nucleatum* OMVs showed no significant changes on viability of PDLSCs.

**(b)** Quantitative analysis of CCK8 assay in (a). The data were based on three independent experiments (n = 3) and presented as mean ± SD.

**Figure S9**


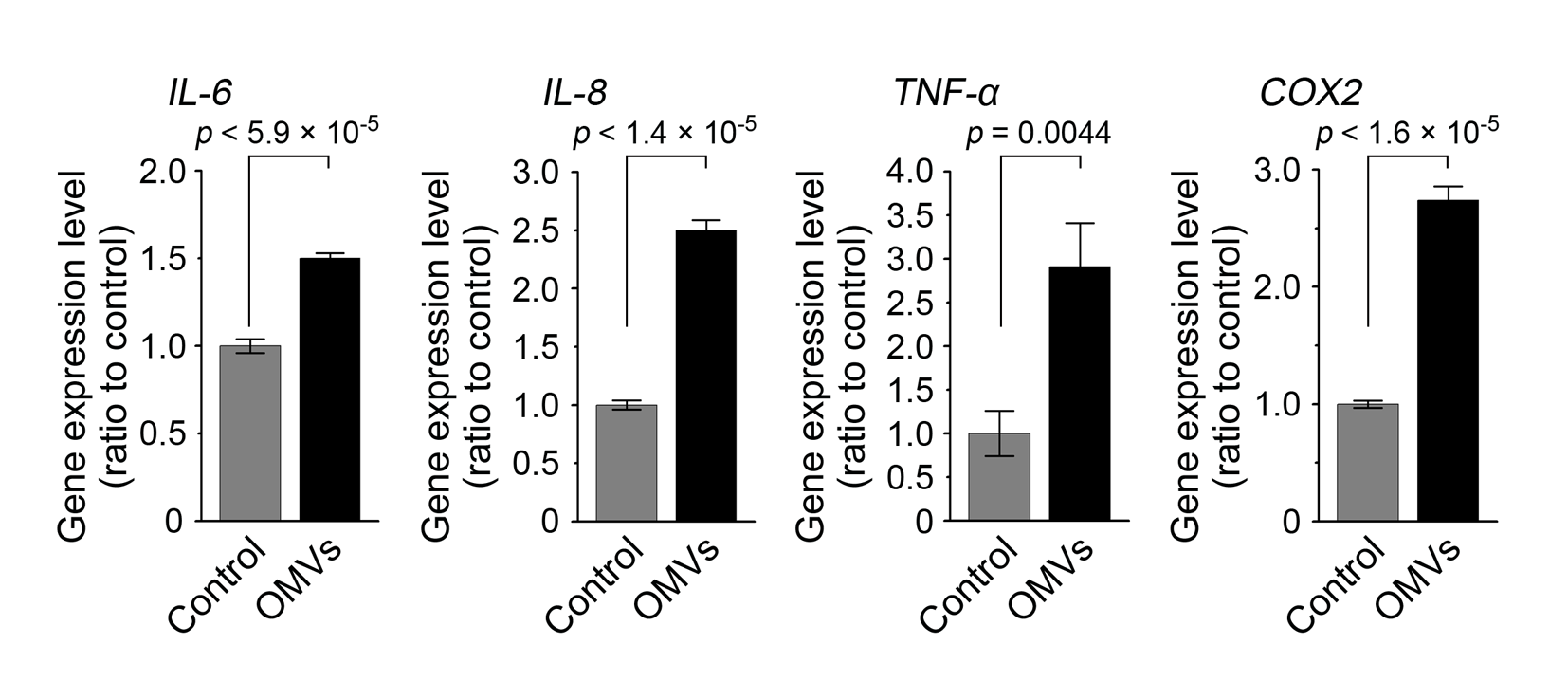


**Figure S9. Gene expressions of IL-6, IL-8, TNF-α and COX2 in hPDLSCs induced by OMVs.** The data were based on three independent experiments (n = 3) and presented as mean ± SD. These data were related to Figure 5b.

**Figure S10**


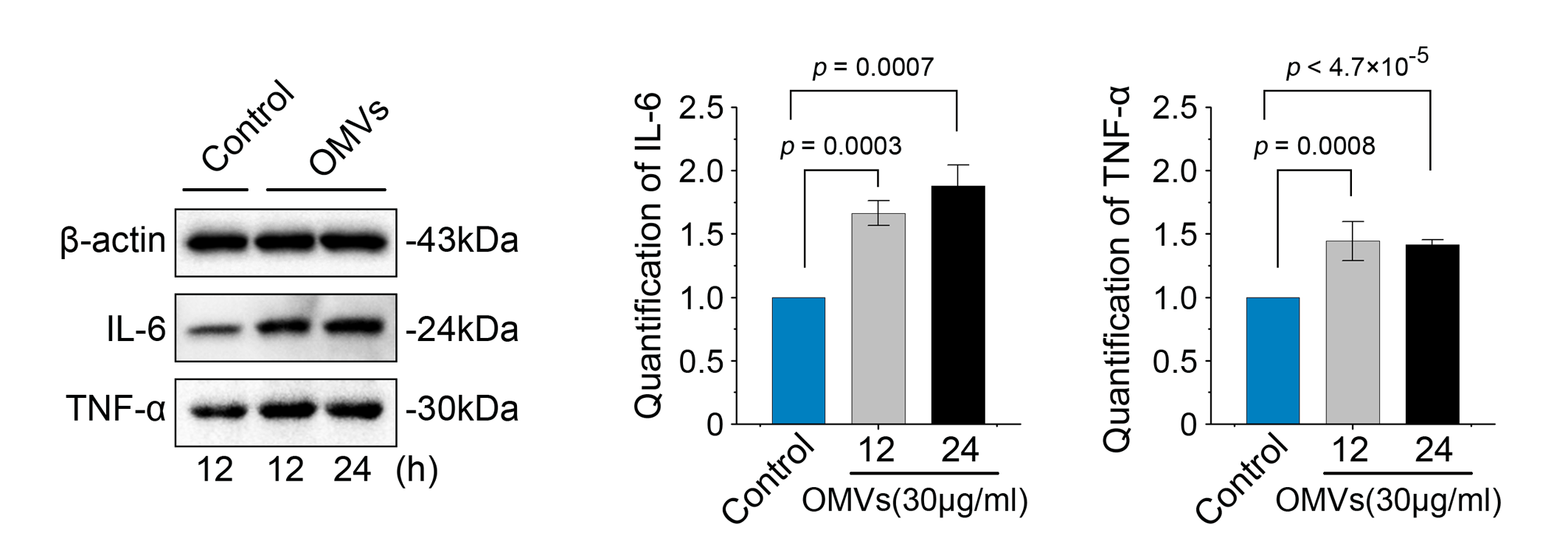


**Figure S10. Protein expression of IL-6 and TNF-α in hPDLSCs induced by *F. nucleatum* OMVs.** Western blotting (left) showing the protein expression of IL-6 and TNF-α in hPDLSCs induced by *F. nucleatum* OMVs. Quantification (right) confirming the change of IL-6 and TNF-α in hPDLSCs induced by *F. nucleatum* OMVs**.**

The data were presented due to three independent experiments (n = 3) and presented as mean ± SD. These data were related to Figure 5h.

**Figure S11**


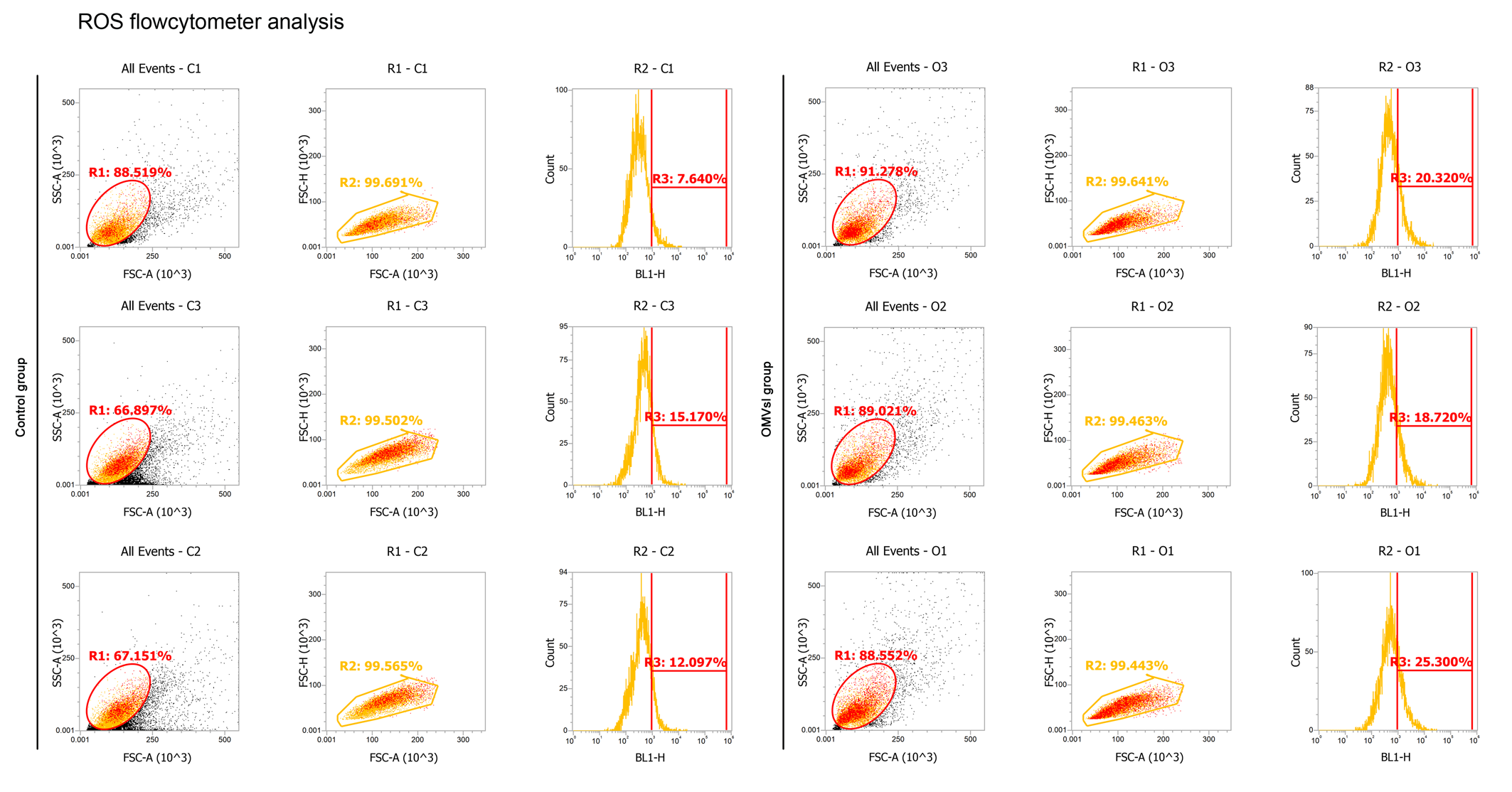


**Figure S11. The original data about flow cytometry analysis showing the increased ROS production in hPDLSCs induced by *F. nucleatum* OMVs and analysis.** These data were related to Figure 5I &5m.

**Figure S12**


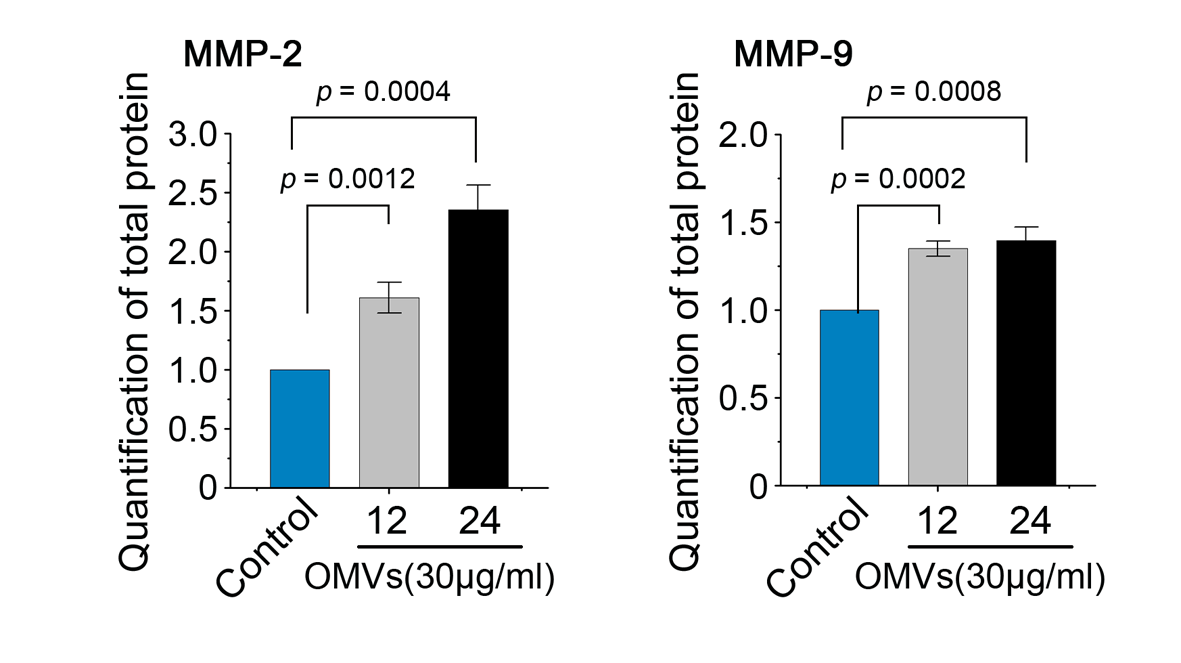


**Figure S12. Quantitative analysis of MMP2 and 9 in hPDLSCs induced by OMVs by western blotting in Figure 5n.** The data were based on three independent experiments (n = 3) and presented as mean ± SD. Significant differences were based on Two-tailed Student’s t Tests.

**Figure S13**


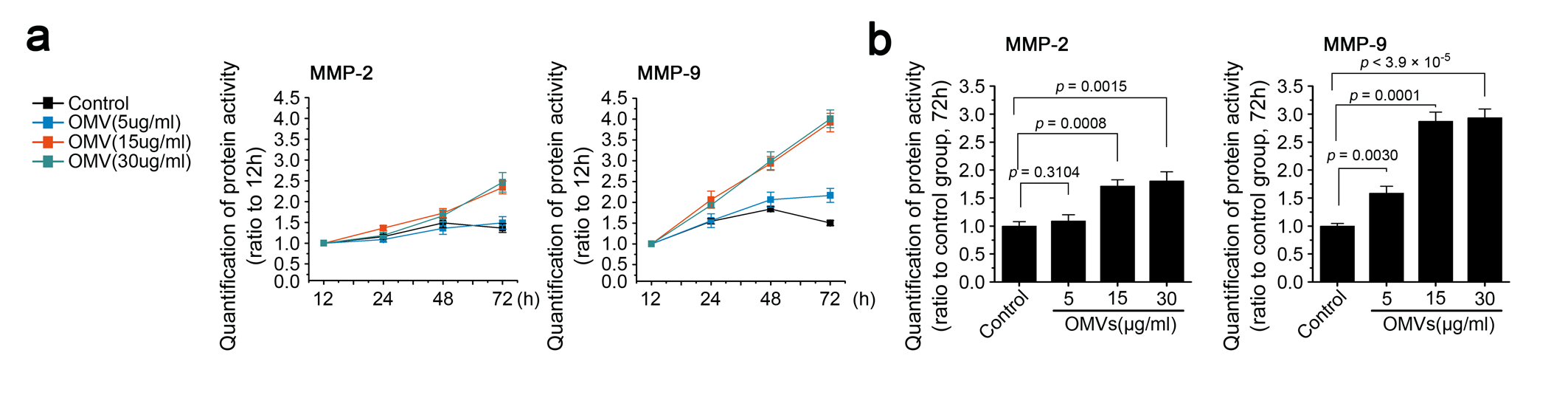


**Figure S13. Activity quantification of MMP2 and 9 in hPDLSCs induced by OMVs in Figure 5o.** (a) Activities of MMP2 and 9 were ratio to 12 h; and (b) Activity quantification of MMP2 and 9 in hPDLSCs induced by OMVs at 72 h. The data were based on three independent experiments (n = 3) and presented as mean ± SD. Significant differences were based on Two-tailed Student’s t Tests.

**Figure S14**


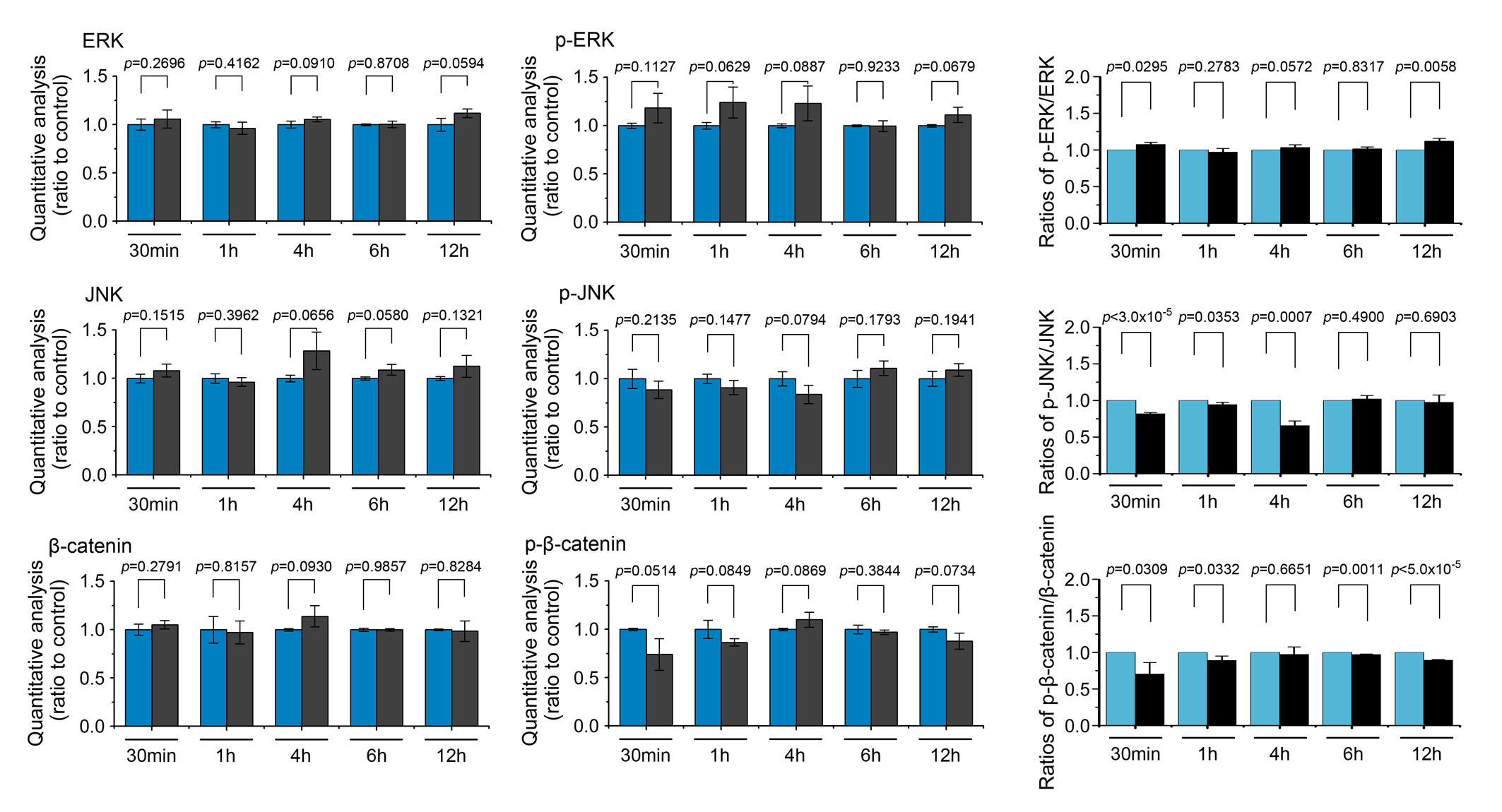


**Figure S14. Quantitative analysis of ERK/p-ERK, JNK/p-JNK and β-catenin in Figure 7c.** The data were presented due to three independent experiments (n = 3) and presented as mean ± SD. Significant differences were based on Two-tailed Student’s t Tests.

**Figure S15**


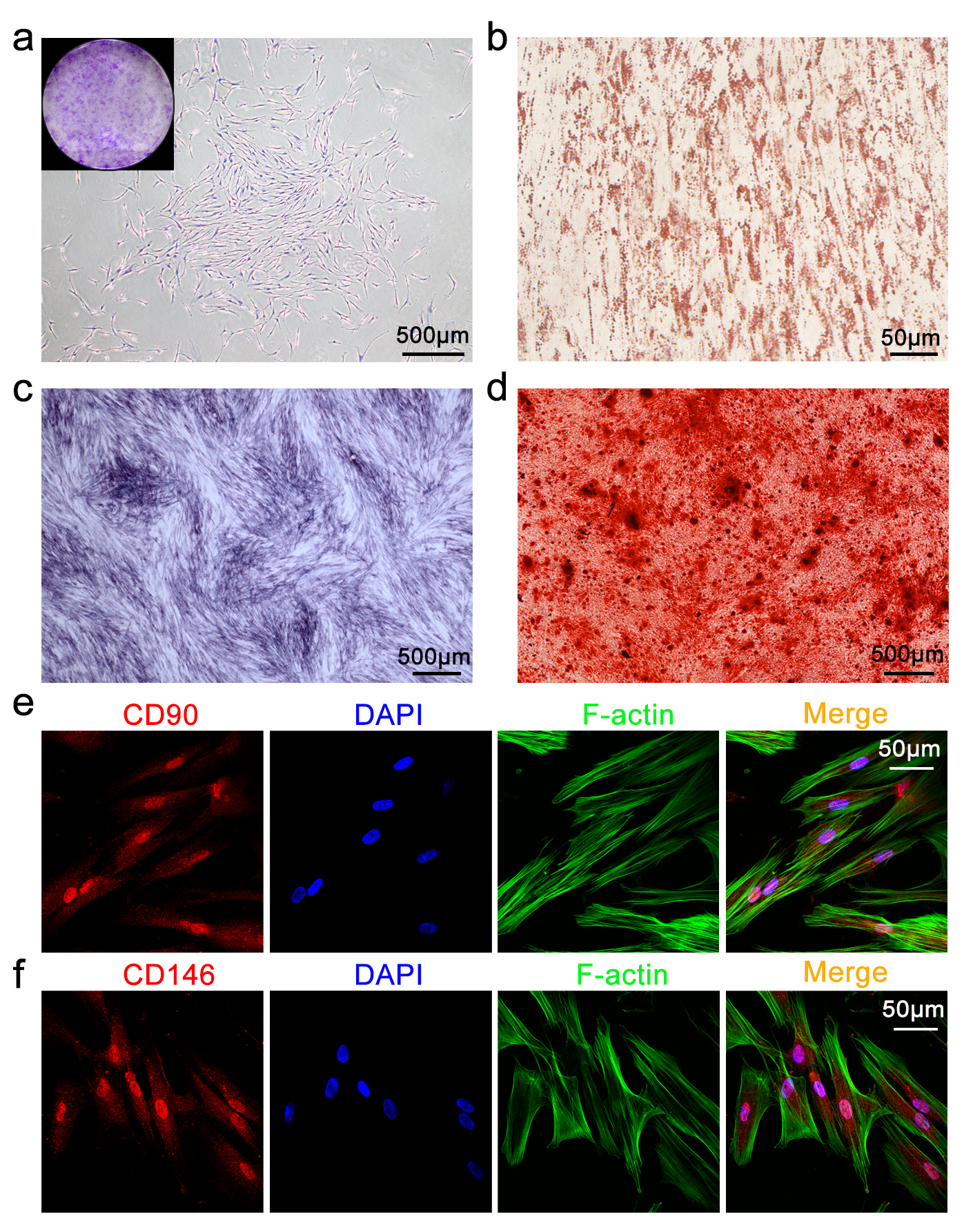


**Figure S15. The characterization of isolated hPDLSCs.**

**(a)** Crystal violet staining showing single colonies formation of harvested hPDLSCs.

**(b)** Oil red O staining showing the adipogenesis capacity of harvested hPDLSCs.

**(c-d)** ALP staining(c) and alizarin red staining (d) showing the osteogenic differentiation capacity of harvested hPDLSCs.

**(e-f)** Immunofluorescent images showing the positive expression of CD90 and CD146 in harvested hPDLSCs. CD90 and CD146, red; F-actin, green; Nucleus, blue. The data were derived from three separate experiments (n = 3).

**Figure S16**


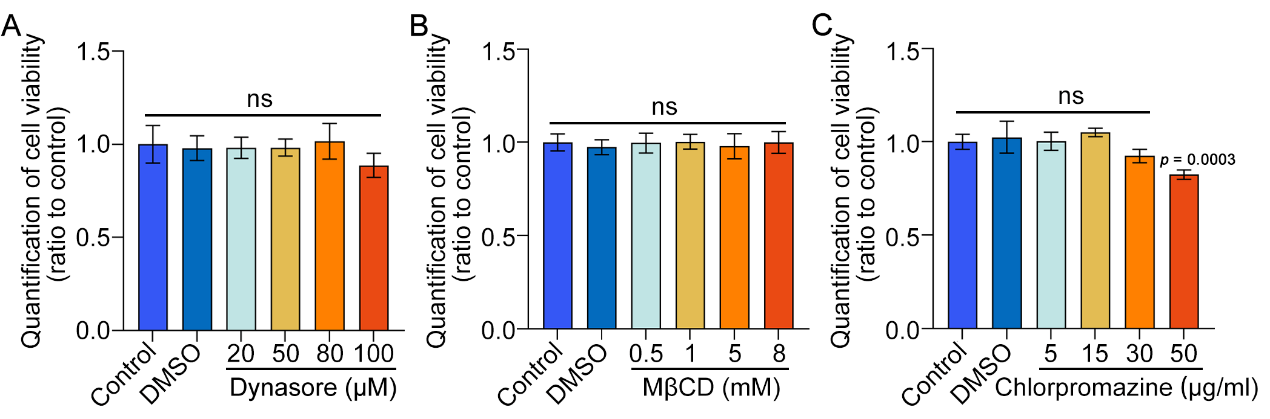


**Figure S16. Effects of dynasore, MβCD and chlorpromazine on the viability of hPDLSCs.**

**(a)** Cell Counting Kit-8 (CCK8) assay showing cytotoxicity of different concentrations (0, 20, 50, 80 and 100 µM) of dynasore on hPDLSCs. Data were derived from four separate experiments (n = 4) and presented as mean ± SD.

**(b)** CCK8 assay showing cytotoxicity of different concentrations of (0, 0.5, 1, 5 and 8 mM) MβCD on hPDLSCs. Data were derived from four separate experiments (n = 4) and presented as mean ± SD.

**(c)** CCK8 assay showing cytotoxicity of different concentrations of (0, 5, 15, 30 and 50 µg/ml) chlorpromazine on hPDLSCs. Data were derived from four separate experiments (n = 4) and presented as mean ± SD.

**Figure S17**


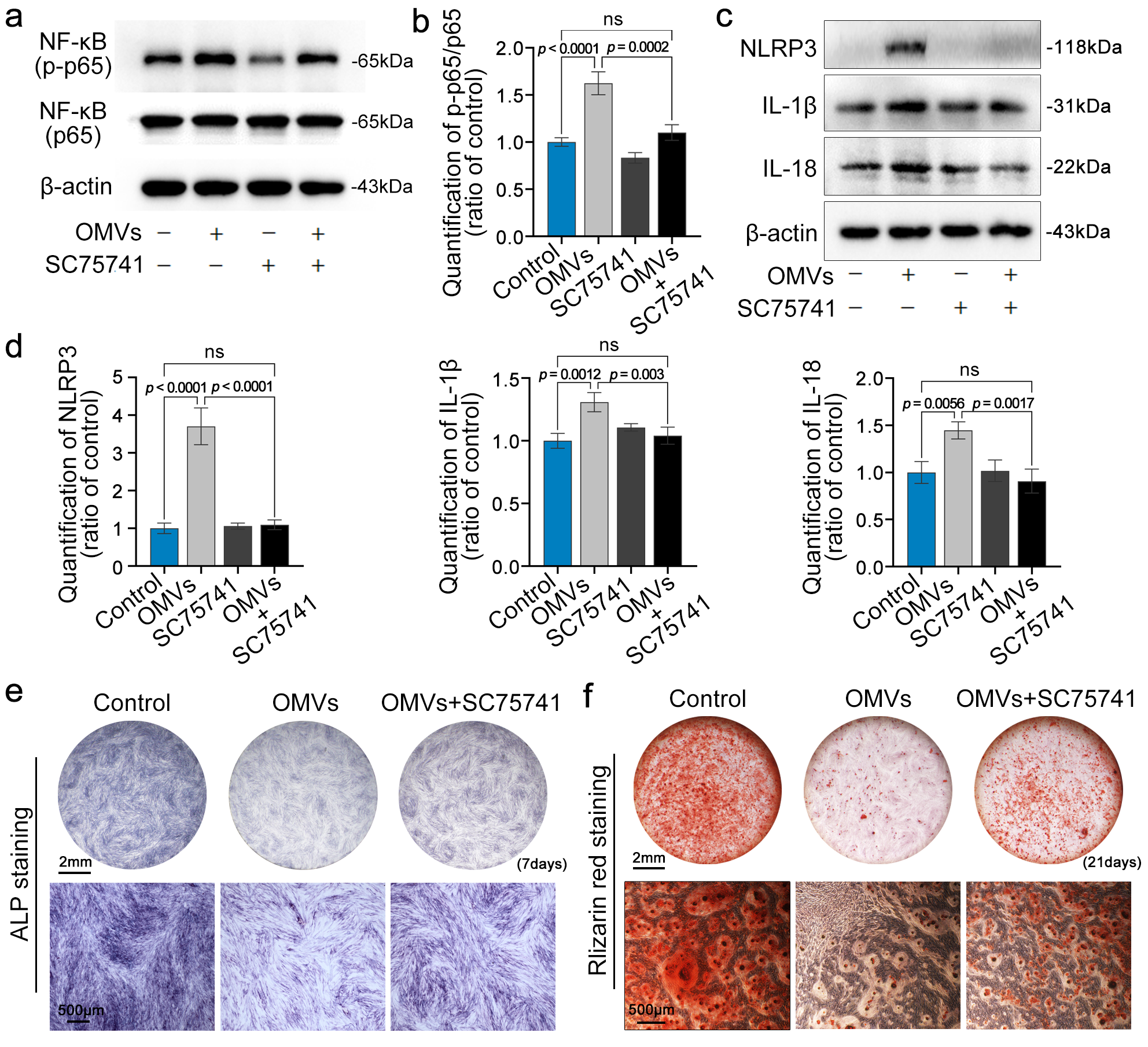


**Figure S17. Inhibition of NF-κB signaling impaired** **OMVs-activated NLRP3 inflammasomes and** **partly restored OMVs-impaired osteogenic differentiation.**

**(a)** Western blotting showing protein expression of NF-κB (total p65 and p-p65) in hPDLSCs pretreated with SC75741 in the presence of OMVs. The images were chosen from three separate experiments (n = 3).

**(b)** Quantification confirming the protein changes in (a). Relative protein expressions were normalized with β-actin. The data were presented due to three independent experiments (n = 3).

**(c)** Western blotting showing that SC75741 (5 μM) both inhibited OMV-induced protein expression of NLRP3, IL-18 and IL-1β. The images were derived from three separate experiments (n = 3).

**(d)** Quantification confirming the protein changes in (c). Relative protein expressions were normalized with β-actin. The data were presented due to three independent experiments (n = 3).

**(e-f)** hPDLSCs were pretreated with SC75741 in the presence of OMVs under osteogenic induction. Then, ALP staining on day 7 (e) and Alizarin Red staining on day 21 (f) were detected. The data were presented due to three independent experiments (n = 3) and presented as mean ± SD.

**2. Supplementary tables**

**Table S1. Primer pairs designed in the** study.

| Protein name | Gene name/gene ID | Primer pairs |
| --- | --- | --- |
| Glyceraldehyde-3-phosphate dehydrogenase | GAPDH  (NM_001256799.3) | Forward: GACAGTCAGCCGCATCTTCT  Reverse: GCGCCCAATACGACCAAATC |
| NLR family pyrin domain containing 3 | NLRP3  (NM_001079821.3) | Forward: TGGCATCGTGAAGTGGTTGT  Reverse: AGCCAAATGCTTACCAGAAAGT |
| PYD and CARD domain containing | ASC  (NM_013258.5) | Forward: TGGATGCTCTGTACGGGAAG  Reverse: CCAGGCTGGTGTGAAACTGAA |
| Caspase 1 | CASP1  (NM_001223.5) | Forward: TCCAATAATGGACAAGTCAAGCC  Reverse: GCTGTACCCCAGATTTTGTAGCA |
| Interleukin 1 beta | IL1β  (NM_000576.3) | Forward: AGCTACGAATCTCCGACCAC  Reverse: CGTTATCCCATGTGTCGAAGAA |
| Interleukin 18 | IL18  (NM_001243211.2) | Forward: TCTTCATTGACCAAGGAAATCGG  Reverse: TCCGGGGTGCATTATCTCTAC |
| Interleukin 6 | IL6  (NM_001371096.1) | Forward: CCTGAACCTTCCAAAGATGGC  Reverse: TTCACCAGGCAAGTCTCCTCA |
| Interleukin 8 | IL8  (NM_001354840.3) | Forward: CTGTGTGAAGGTGCAGTTTTGC  Reverse: AACTTCTCCACAACCCTCTGC |
| Tumor necrosis factor | TNFα  (NM_000594.4) | Forward: CCTCTCTCTAATCAGCCCTCTG  Reverse: GAGGACCTGGGAGTAGATGAG |
| Prostaglandin-endoperoxide synthase 2 | COX2  (NM_000963.4) | Forward: CCACCCGCAGTACAGAAAGT  Reverse: GGCAGGATACAGCTCCACAG |

**3. Supplementary information about promoter bindings**

**(1). NF-κB bindings at the promoter sites of NLRP3 gene**

-4000~+200bp

TTTTTTTTTATATTTTTAGTAGAGATGGGGTTTCGTCATGTTGGCCAGGCTGGTCTCAAACTCCTGACCTTGTGATCTGCCTGCCTTGGCCTCTCAAAGTGCTGGGATTACAGGCATGAGCCACTGCACCCAGGCTGAGCTCCTTTCTTTTGGAGCATATTTGGTCTGCTTTCTCTCTAGCTTCAGCACCTGAAGTTTTTGCTGCTCCTGCCTCAGCTCCAGCCCCGAATGACACCTTTCACCTGGTTCATGGAGACTCAGGTCTGCTGTCATGCCCCTAAGCAGGACTAGATGCCTCTCTCATGTTGTAGAGCATTCTCTCTGCAGTTCTGTAGAAAGGTGGTAGTCATCGGAACCAACTGCTTATTCTAGCTTCTCTGTGCCAGGCTCTGTTTTAAAGGCCTCACGTTTAGCCATTCACTCAGTGTTTTCTGACAGCCTTCTAGGATAGGTACCGTTATTTGGGTTGATGTAGTGCTTGGTCAAATGCAGTATAATGAAGGGATCAAGGAGGGGGCAATTCCAAGCAAAACCACTTGTTCATCCATCCTGAGTCTAGGAGATTTGGAAGATCTAAAGAAGTAGCAGGCAATTATTGTCACCCTTTTACAGGTATGAGGCACAGACAGACCCAGTCATTTGCTGACATCCCTCCACTGAACTGGGTGCTCGTCCTTTTCACTCACCCAGAGGCTGAGCGAGTGCCTCTCACCCACCAAGCTCTGTTGAACTTGTGCCTTTGCCGGTCTGTGGTCCTGCGGCCGTCTCATGAGCTGCAGAGTAGGTCTGTGTGTGTCTCTCCTCAAGCTACTCAAGCTGAGGCTTTGTGTGCATGCCTGCCACATACCAGCCATTCCGTGAGTGTTAGTGGAACAGGTGAGTCAATGAGTCAGGGAGTAAAATTTGTGCTTTAAAAGTAGAACATGTCGGAGAGAACCTGTACTGCCTTCCAGCCATCTTGCTGTGAGAGGACAGGGCATGGACTCTGGAGTTTGAAGGCTGGGTCAGGTCATGCTCTGGATTCTTTACTCACTCGCATGGCATGTCCTTAGTTTCACTTCCCTCACTCTCAGTGGAGCCGTTGCAGCGGGCAGTGTCAGAGGCAACATCCATTTAGCACTGCCGATTTGACCCCCAGCCCCATCTCATACTGGCCTCCCACCCTGCTTCTGTGGGGTGCCTCAGAGGAGAGCAAGGTGAACCCCAGTATGGAACCGAGACACGGTTTTGACAACACTGTTGATCCCATTTGGAAAATCCAGCTATGTGATTAACCATTAGTCTCTCTGCCTCTGCTCTGATGTAAGTGGAGACCACATCCTTCCTGCCCCTTCTGGGGCTGCGACTGCTATAAATTCATTGCATTTCCTCTCTAGCTGTTCCTGAGGCTGGCATCTGGGTAAGTCCAGCTCCGCGGGGCAGTGTGGGCGACAGGACCGGGAGAGCAGTGGGGGAGATTGTGTTCAGAGGGCAGAACTTGTCCTGGTTAATGCACTAGGAATTTCTTCTGGCGTCTCCAAGGTTGTGCTTCAACAACATTTCTGGAATGTTCTTTTATCATTATTTGGGCTCTACGTGTGCAAGTTCGTGTGTGTGTATGTGGCGGGGGGAAGCGGGGAGGACAGCGGCAGCAACCATAGTTTACACCCACGACTTCAGCAGATTTGCCCAGTATCCCTCCCAGGGGAGAAGATGGATGGGATATACAGAAGGAAACAAACCTCCTTAAAGACCAAAGGGCAGCAGATTTTTGTGCTGGTGTTAGGGTGGAAGGAGCACAGAAGAAAGGCAGAATCCTGATGCAGATGGAAGGACTGATGGGGCAGGAGCTGGGAGGGTATGCGGAGCTAATAGGGCTGTCTGGAGAGCAGTCACCTCAGGATGGGCAGGAGCTGGGAGGGTATGCGGAGCTAATAGGGCTGTCTGGAGAGCAGTCACCTCAGGATGCACAGGGCTGGGTTTGCAGGGCTCAGGAGGCGAGGAGTAGATAGGCAGGAATGGAGATGGGACGGGAGAGATGAGTGAGTCTGAGGCATCTTGACATTGAACTGCAGTCACAGAAATTCCAATTTGGAGAGCTCCACCGTTTTCCAGGCTCTCTGCCTCCCTTGAGTGTTATTTATGGCTTTAGGTTGAGGTGCTTTGCCAAATAGAGTATAATCAAGGGATCAAGGAGGGAACAATTCCAAACAAAGCCAACTTGTTCATCCATCCGGACTCTAGGAGATATGGAAGATCTAAAGATACAGCAGGTGATTGCTAGTTTTTGTGGAACTCACAGTCTAGTTGGGAAGACTATGTTGGTGAATGCTGCAGTGATACACCTGTGATAAATGCTGGGGAAGTGTGTCTTTTAGTCATCTATTTTGTTGTCTTTGTCTTTGTCTGACTGAAACAAGTGATGGAAGACATGGTCTCATATCTCTGGTCAAAATCCTGGCTCTGTTCCTTCCTGGTTCTAAACCCCTCGGCAGGCTTCCTAGTCCCCCTAAGACTCAGTTTATGCATCTATAAAATGGGGCTAGGAATAGGTGCACCTCATAGAGCTCTGTGCAGGTTAGATGGGGAAAAACATCAGTGAGTGCCTAGCCTGTGGAAAGCTCTAAATTAATGTGAATTATAATGATAACAATTGCGAATTTTGCAAGGAGACATTAAAGGATGTATGTTTTTATTTATTTTATTTTATTCTATTTTATTTTTTGAGATGGAGTTTTGCTCTTATTGCCCAGGCTGGAGTGCAGTGGTGTGATCTTGGCTCATCGCAACCTCCGCCTCCTGGGTTCAAGCGATTCTCCTGCCTCAGCCTCCCGAGTAGCTGGGATTACAGGTGCCTGGTACCATGCCCGGTTAATTTTTGTATTTTTAGTAGAGACGGGATTTCTCCCTGTTGTTTGGTGAGCCTGGTCTCCAACTCCTGACCTCAGGTGATCCGCCCACCTCGGCCTCCCAAAGTGCTGGGATTACAGGGATGTATGTTTTTATTATTCTCCATTATGCACTCCCAGCTTCATCTCACTTCAATCCACTGGTTGATAGAAGTGCAGTCAAAGACTGTCCCCTCCTCTGCAAGCTTTTATGTTCTCTCTCCTCTCTCCCTAGCCACGTCCTGTTCTGTTATCAACACACTTACTCACCTCCTGGCCTTCTCCTTCTATTGTCTCTCATCCCTCTTCCCTCACAAAAACAGAAGCAAAGAGCCAGAGCCTTCAGTTTGGAGGAACTGAAAACATTCTCTTCTGCTTTCTCATTTTGTAGATGAGGAAACTGAAGTTGAGGAATAGTGAAGAGTTTGTCCAATGTCATAGCCCCGTAATCAACGGGACAAAAATTTTCTTGCTGATGGGTCAAGATGGCATCGTGAAGTGGTTGTTCACCGTAAACTGTAATACAATCCTGTTTATGGATTTGTTTGCATATTTTTCCCTCCATAGGGAAACCTTTCTTCCATGGCTCAGGACACACTCCTGGATCGAGCCAACAGGAGAACTTTCTGGTAAGCATTTGGCTAACTTTTTTTTTTTTGAGATGGAGTCTTGCTGTGTCGCCTAGGCTGGAGTGCAGTGGCGTGATCTTGGCTCACTGCAGCCTCCACTTCCCGGGTTCAATCAATTCTCCTACCTCAACTTCCTGAGTAGCTGGGATTACAGGCGCCCGCCACCACACCCGGCTCATTTTTGTACTTTTAGTAGAGACACAGTTTTGCCATGTTGGCCAGGCTGGTCTTGAATTCCTCAGCTCAGGTGATCTGCCTGCCTTGGCCTCTCAAAGTGCTGGGATTACAGGCGTGAGCCACTGTGCCCGGCCTTGGCTAACTTTTCAAAATTAAAGATTTTGACTTGTTACAGTCATGTGACATTTTTTTCTTTCTGTTTGCTGAGTTTTTGATAATTTATATCTCTCAAAGTGGAGACTTTAAAAAAGACTCATCCGTGTGCCGTGTTCACTGCCTGGTATCTTAGTGTGGACCGAAGCCTAAGGACCCTGAAAACAGCTGCAGATGAAGATGGCAAGCACCCGCTGCAAGCTGGCCAGGTACCTGGAGGACCTGGAGGATGTGGACTTGAAGAAATTTAAGATGCACTTAGAGGACTATCCTCCCCAGAAGGGCTGCATCCCCCTCCCGAGGGGTCAGACAGAGAAGGCAGACCATGTGGATCTAGCCACGCTAATGATCGACTTCAATGGGGAGGAGAAGGC

**(2). NF-κB bindings at the promoter sites of CASP1 gene**

-4000~+200bp

TAACATTATGCACATTTAAAATATTACTTATAATAGAATTGTTACATTCATTTATTTTGAGATGATTTTACCTTTGTAATTCTATTTTCCAAGTACACATTTAAGTCAACAATTTGGTTCCTATAAATTATATCCACTCTGTCTACAATTCCCCATCCAACTTGGGTGGTTGGTAATTACAACTCAGTCACTATAAGAACAACTTCCTCTTACCAAAATTTTTACAAATTTGTGGAGTGTGTCCAGCCAGAAATCAACAAAAAAATATATATTATTACAACCACTGACCATTTTCGCTTCAAAATTTTAATGTTATTTTTGTATAATTTACGTCAAAGAAAATGTACTCCTTTTAAGAATACAAGTTGATAACTAATGATACATGGCTATTCTAGTATAATAACATCCTAGTGAATAGGCAAACCGATTTTGTCACCCCAGAAAGTTTTGTCACGCCCATTGTTATCAATCTCCTTCCACTTCCTCCTCAGATAAGTAGTCATCGAATTTCTATCACTGTACGTTAGTTTTTCCTAATCTAGAACTTTATATAATTGGAATCACAGGGTATGTGTTCCTTCCTATATGGCTTCTTTCACTCATAATTGAAACATATTTTTTTCATCCATGTTATTGCATATATTAGTAATTTTTCCCTTTCATGACTGAATAGCATTTCAGTGTACATGTATAGCACAAATTGTTATTTTATGCTCTGGAATTGGATATTTATTTGTTTCCAGTTTTTGACATCTTCATTTTTGAGGAAACAGTGGGCCCTTGCAATCTAGTGGCTCAGTGTTTAGCTTTCCATGACATGAGACTCAAGGCTTATGGTTCTACATTCCCAGATATATGGTCTAGTCATGGCTTCTGTTTCTGGGATGTCACTCTGACATGCTTCAACTCAGTGGTTCATGGCTCTGGCTGTGGGCGAAACCCTCATGTGAAATACTTTAGTTAATAGAAAAAAAATTGAATCATTTAAAAGTACATGATGGGAAGATAAGTAATACACAGGCAATTTCTGCTTTCTTCCATGCTACAAATCCTCCCAGTGGCAAGGAGCACAAGTTTGTGCTGCCATCGGATTAAGTCATTGGCAAAGTAAAGATAAAACACCATGAGAGGTTGGGCATGGTGGCTCACATCTATAATCCCAGCACTTTGGGAGGCCAAGGTGGCCAGATTACGAGGTCAGGAGTTTGAGACCAGTCTGGTGAACATAGTGAAACCCCATCTCTAGTAAAAATACAAAAAATTAGCCAGGTGTGGTGGTGCCTGTAATTCCAGCTACTTGGGAGGCTGAGGCAGGAGAATCACATGAACCCGGGAGGCGGAGATGGCACCATTGCACTCCAGTGGGGTGACAGTGCGAGACTCTGTCAAAAAATAAAAAATAAAAAAAAAAAACGATGTCCTCCTACACAGTGAGTTCTAGTCAAACCCAAACACATTTAACCCTTCACCTAGAGCACAGAAAATAAAACTGGTAGCCCACATGTCAAAAGGTGGATGTTTTTCTAACTCTGGGTCTTTAATCTCCAAAAATATTAAGGTAACAAATCAGGAGAATTTTTGGTAAAGAATTTGATAAACGTAACATACCGTGTGCATTTTGCAATTAGCCCACCTTTCATCAAATAGATTTATAAAATAAATTAGCCACATTGAGTTTGTTTCTGGGATAAGCAATTGAACATGTGATTGCAAAGGCAGAGACCTTTTCTGGCCTTCCCTGGCTCCTCTGTCTAAAGTTCATGAGTCCTGCAATATTTTGTCTCCTACTTTGCCAATTTTTTTTTTATCATTCTCATCACTTGCTACATTTGAACAGTCTGAATATTCCAGTTACATATTACATACCAGGTGTATTAGTCTGTTCTCATGCTGCTAATAAAGACATACCCAAGACTGGGTATTTATAAAGAAAAAAAGAGGTTTAATGGGCTCATAGTTCCACATGGCTGGGGAAGCCTCACAATCATGGTGGAAGGCAAAAGAGGAGCAAGGACACATCTTCCATGGAAGCAGACAAAAAGGCATGTGCAGGGGAACTCCCCTTTATGAAGCCATCAGATCTCATGAGACTTTTTCACTATCATGAGAACAACATGGGAAAAACCCACCTCCATGATTCAATTACCTCCCACCAGGTGCCTCCCATGACACCAGGGGATTATTACAATTTAAGGTGAGGTTTTGGTGGGGACACAGAGCCAAACTCTGTCATTCTGCCCCTGGCCTCTCCTAAATGTCATGTGCTCACATTTCAAAACCAATCCTGCCTTCCAACTGTTCCTCAAAGTCTTCACTCATTCCAGCATTAACCCAAAAGTCCAAGTCCAAAGTCTTCTCTAAGACAAGGCAAGTCCCTTCCACCTATGAGCCTGTAAAATCAAAAGCAAGTTAGTTACTTCCTAGATACAACGGGGATACAGGCATTGGGTAAATACACCCATTCCAAATGGGAGAAATTGGCCAAAGGAAGGGGCTACATTCCCCATGCGAGTCCAATATCCAGTAGGGCAGCCAAATCTTAAAGCTTCAAAATGATCTTTGACTCCATGTCTCATATCTCGGTCATGCTGATGCAAGAGTCGGGGTCCCATGGCCTTGGGCAGCTCCACCCTCGTGGCTTTGCAGTGTACAGCATCCCTCCTAGCTGCTTTCATGGGTTGGCATTGAGTGCCTGAGTATTCCAGGTGCATGGTGCAAGCCGTTTGTGGATCTACCATTTTAGGATCTGGAGGATGGTGGCCCTTTTCTCATGGCTCCACTAGGCAGTGCCCCAGTGAGGACTCTGTGTGGGAGCTCACACCCCACATTTCCCTTCTGCATTGCCCTAGTAGAAGTTCTTCATGAGGGTCCCACCCCTGCAGCAAACTTCTGCCTGGACATACAAGCATTTCCATACATCCTCTGAAATCTAGACAGAGGTTCCCAAACCTCAATTCTTGACTTCTGTGCCCCTACAGGCTCCACACCACATGGAAGCTGCCAAGGCTTGGGGCTTGCACCCTTTGAAGTCACAGCCCAAGCTATTTCTTGGCCCCTTTTAGCCATGGCTTCAGTGGCCCGGGAAGAAGGGCACTAAGCCTTTAGGCTGCATACAGCAGAAGGGCCCTCGGCTTACCTCATGAAAACATTTTTTTCCCTCTTAGGTCTCCAGTCCTGTGATAGGAGGGGCTGCCTGGAAGGTCTCTGACATACTCTGGAGACATATTCCCCATTGTCTTGGAGATTAATGTCTTAGAGATTAACATTTGGCTCCTCGTTACTTCTGCAAATTTCTACAGTTGGCTTGAATTTCTCCTTAGAAAGTGGGTTTTGCTTTCCCAGCACATCATCTGGCTGCGAATGTTTCAAACTTTTATGCTCTGCTTCCCTTTTAAACATAAGTTCCAATTCCAAACCATATCTTTGCAAATACATAAAAATGAATGCTTTTAACAGCACCCAAATCACTTCTGGAATGCTTTGCTGCTTAGAAATTTCTTCTGCCAGATGCCCTAAATCATCTCTCTCAAGTTCAAAGTTCCACAGATCTCTAGGGCAGGGGCAAGATGCCACCACTCTCTTCACTAAAACGCAGCAACAGTCACTTTTATTCCACTTCCCAACAAGTTCCTATCTTCATTCGAGATGAGCAGCATTTAGTCAAAGCCATTCAACAAGTCTCTAGAAAGTTCCAGATTCTATTTTCCTATCTTTCTCTAAGCCCTAAGAACTGTTCCAACCTCTGCATTTTACCCAGTTCCAACATCGCTTCCACATTTTTGGAGATCTTTACAGCAGCACTCCATTACTCAGTATAAATTTACTGCATTAGTCATTTCTCACACTGCTAATAAAGACATACTCAAGGCTGGGTAATTATATAGAACAAAAAAGATATTTAATAGACTCACAGTTCCACATGGCTGGGGAGGCCTCAGAATCATAGCAAAAGGCAAAAGAGGAGCAAGGACACGTCTTACATGGGTGCAGGCAAGAGGGTATGAGCAGAGGAATTCCCTTTTATAAAACCATCGGATCTCATGAGACATTCATTATCAAGAGAACAGCATGGAAAAAAAACAGCACCCATGATTCAATTACATCCCACCAGATACCTCCCATAACACATGAGGATTATTACAATTCAAGGTGAGATTTGAGTAGGGAAACAGAGACAGACCATATCACCAGCCTTCATGT

**(3). NF-κB bindings at the promoter sites of ASC(PYCARD) gene**

-4000~+200bp

ACTGCACTCCAGCCTGGGTGACAGAGCAAGATTCTGTCTAAAAACATATATATTACAAAAATTGGCTGGGCATCGTGGTGCACACCTGTAGTATCAGCTATTTGGGAGGCTGACATGGGAGGATCGTTTAAGCCCAGGAGGTCAAGGCTGTAGTGAGCTATAATCATGCCATTGCTCTCCAGCCTGGGCAACAGACTGAGACCGTATCTCAAAAAAATAAAAAAAAAAAGAGAGAGAAAAAGAAAAAGAAAAGAAAGGAAGGAAGGGAAGAAGGGAGGGAGGGAGGGAAAGGAAAGAAAGAGAGGAAAGAAGAAAGAAAGAAAGAAGAAGGAGGAAGGAAGGGAGGGAGGGAGGGAGCAAGGGAGGGAGAGGGGGAGGGAGGGAGGGAGAGAAGGAAGGAAGAAAGGAAGGAAAAGAAAAGAAAGAGAGGAAAGAAGAAAGAGAGGAAGGAAGGAAAGGGAAGGGAGGAAGGAAGGAAAGAAGGAAAGGAGGGGGGAGGAAGGGAGAGAAGGAAGGAAGGAAAGAAAGAGAAAGAAAGAGACAGAAAGGAAGGAAAGAAGGAAGGAAGGAAGGAAATAAAGAGGAGATAGAAAGAGAGAAAGAAGAAAGAAAGAGGAGGGGAGGGAGGGAGGAAGGGAGGGAAGGAAGGAAGGAAGAAAGGGAAAGAAAGAAAGAAAGGAAAGAAAGAAAAAGAAAGAAAGAAAGAAAGAAAGAAAGAAAGAAAGAAAGAAAGAAAGAAAGAAAAGAGAGGCCGAGTGTGGTGGCTCATGCCTGTAATCCCACTAATTTGGGAGGCAGAGGCGAGCAGATCACAAGGTCAAGAGATCAAGACCATCCTGGCCAGCATGGTGAAACCTCATCTCTACTAAAAATACAAAAATTAGCTGGGTGTGGTGGTGCGCACCTGTAGTCCCAGCTACTTGGGAGGCTGAGGCAGGAGAATCGCTTGAACCTGGGAGGTGGAGGTTGCAGTGAGCCGAGATCACGCCACTGCACTCCAGCCTGGCAACAGAGCAACACTCCATCTCAAAAAAAAAAAACAGAGAGAGAGAGAAAGGGAAAAACAAAAGAAGGAAAGGAGGGAAGGAAGGAAGGAGGCATGCGAAGGACTGCAGACTAAAGGCTTATTTCTATTATTTATTAGAAGAGGTGACTCCTAGGTTGGGTGTGTTGGCTCACACCTGTAATCCCAGTGCTTTGGGAGGCCAAGGCAGGAGGATCGCTGAGCCCAGGTGTTGGAGATCAGCCTGGGCAACATAGCCAGACCCCATCTCTACAAAAAAATTAAAAAATTAGCTGAGCATGGTGGCATGCACCCGTAGTTCCAGCTACACAGGAAGCTGAGGTGGGAGGATCGCTGGAACCCAGGAGTTCGAGGCTACAATGAGCTATGATTGCGCACTGCACTCCAGCTTGGGCAACAGAGTGAGACCTCATCTCTAAAATAAAAGCAGAAAAGAAAAAGTGATGCCTGATGAACGTCAGAAGACAGGCAGGAACTCACCAAGTAGACCAGGAGGAAAAAGAGGCCAAGCAGAGAGCAGCTCCCACAGGGGCGGAGGTTCACGGATCAGCAAGCTGGAAGGGGCAGCTGAAGAGGTTGGCAAGGACCTTGAAGGCCAAGCCAAGGGCTTGGGACTTGAGTTTGAGGGCACTGGGGAGTCATGAGGGGGTCTCTGAGCCAGGCAGGTGCAGGATCATATGTGTGGGTTAGGAAGATGTATCCTTCTGGCCACAGTGCACAAAAGGAAATGGAGATTCTGGAAGGATAAAGGCCAGTGAGAAGGCATGATAAACATCCATGAGCCAAGGAACACTGGCTGTTGGACCACAAGTGCCACCCATGGTGAGGATGTTGGCACACAGCTTACCCCAGCCCACATGCTACAAGGATGGGGAGCAAGGCCAGGCACAGTGGCTCACACCTGTAATCCTAGCATGTTGGGAGGCCAAGGCAGGAGGACTGCTGAGCCCAGGACTTTGAGACCAGCCTGAACAACATAGGGAAACCCATTCTCTATAAAAATTTTAAAAACTAGCCAGGCATGGTGGCACAACTGTAGTCGCAGGTACTCAGGAGGCCGAGGTGAGAGAATTGCTTGAGCCCAGGAGTTCAAGGCTGCAGTGAGCCACAATCCCTCCGGTGTACTCCAGCCTGGGCAACAGAGTGAGGTCCTGTCTTCAAAAGAAAAAACAAACAACAACAACAACAACAACAAAAAGGCTTTCAGCAGGGTAGCAGAGCACTGCTCCCCATCGTGGTTTTTGTTTGTTTGTTTTTGTTCCTGTTTTGAGATGGAGTTTCGCTCTTGTTGCCCAGGCTGAAGTGCAATGGTGTGATCTCAGCTCACTGCAACCTCCACCTCACGGGTTCAGGCGATTCTCCTGCCTCAGCCTCCCCAGTAGCTGGGATTACAGGCACCTGCCACCACGCCCGGCTAATTTTTGTATTTTTAGTAGAGATGGGGTTTCACTCTGTTGGCCAGGCTGGTCTCAAACTCCTGATCACCCACCTCGCCCTCCCAAAATGCTGGGATTACAGGCATGAGTCACCGTGCCCGGCCCATCCTGGTTGTCTTGTTTGTTTTTGAGACAAGGTGTCTGTTGCCGAGTGGCACAGAGTGCAGTGGCACAATCATAGCTCACTGCACCCTCAACCTCCTGGGCTCAAGTGACCCTCCGGCCTCAGCCTCCCAAAGCACTGAGATGACATGCGTGATGAGCCACTGCACCCAGCCATGCTGGAAGCTTTTGAATACAACATGCTGCAGGCATCACAGCCTCATTCATTCCTTCACTCAGCAAATCTTTACTCAGCACCTAATGTGTTCCAGATACATTTTTTTTTTTCAGATGGAATCTAGCTCTGTCACCCAGGCTGGAGTGCAGTGGTGCAATCTTGGCTCACTGTAGCCTCTGCCTCCGAGGTTCAAGCGATTCTCATGCCTCAGCCGCCCTAGTAGCTAGGATTACAGGCGCCCTCCACCACACACAGCTATTTCCAGGTACATTCTTGACGCTAGGAATTCAGCAAAGAATAAGACAGTTAAGGTCTCCGATGCTCATAGGCCTCACATTTTAGAGAGGGATGAATGTCCAATAAGCATATAAACGTATAATATGTCAGGGTCGTATGACTACAAGGAACAGTGATTGTTACAACCCAGATGAGAGGGAAAAATAAAGGATTCCAAATATCCCCCTTGGGAAGTAGAGTCAGGATTCAAACAAAGAACTGTATGGCTTCAAGTTCATGGTCTTTAATCTCCTGGAGGCTGTCTCTCTTTCTTTTTTCTTTTTTTTAATCAGTGTTGGGATCAAATTCTGGCTCCCCTAGGAAGCATCTGGCAAGGTTTCGGGAGCCATCGGGTTGGCCATGTTATGCTGGAATATTTATAAGCACCGGAGGGTTATCCCCATGTCGTAGAAAATGAAACTGAAGCTCAGAGAGATTTGCACTCTCTGCCCTTTTGTACAACTCATTTTTCCCCAGTATGTGGAATTGAGGGAGCTTCACGCTTCTAGCTGTCATGATTCCAAGATTCTACGACATGTGGGAGAGGATCCTAAGGTTCGGGGAACCGCGGAGGTTTCGGGGTTCTAGAAATCCGAGGTTCTAAGCCTAGGTGCTCCAATAAACCCAGTGAGAGCCAGCCCAGGTTTCCGGTCTGTACCCGCTGGTGCAAGCCCAGAGACAAGCAGGCGCCACCCATGAGCCCCTCTGCGGCCCCCTCCCGGGTCCCACCTCGCAGGCCAGCTGGAGGGCGCGATCCTGGCGTCCCCCGACGGCCTGGGGCCCCAATCCAGAGGCCTGGGTGGGAGGGGACCAAGGGTGTAGTAAGGAAGCGCCTTTTGCTGGAGGGCAACGGACCGGGGCGGGGAGTCGGGAGACCAGAGTGGGAGGAAGGCGGGGAGTCCAGGTTCCGCCCCGGAGCCGACTTCCTCCTGGTCGGCGGCTGCAGCGGGGTGAGCGGCGGCAGCGGCCGGGGATCCTGGAGCCATGGGGCGCGCGCGCGACGCCATCCTGGATGCGCTGGAGAACCTGACCGCCGAGGAGCTCAAGAAGTTCAAGCTGAAGCTGCTGTCGGTGCCGCTGCGCGAGGGCTACGGGCGCATCCCGCGGGGCGCGCTGCTGTCCATGGACGCCTTGGACCTCACCGACAAGCTGGTCAGCTTCTACCTGGAGACCTACGGCGCCGA
